# Developing a Set of Key Principles for Care Planning Within Older Adult Care Homes: A Modified Delphi Survey

**DOI:** 10.1111/hex.70433

**Published:** 2025-09-29

**Authors:** Jonathan Taylor, Thais Caprioli, Jacqueline Damant, Yuri Hamashima, Nick Smith, Madalina Toma, Michele Peters

**Affiliations:** ^1^ Applied Health Research Unit, Nuffield Department of Population Health University of Oxford Oxford UK; ^2^ Department of Primary Care and Mental Health University of Liverpool, Waterhouse Building Liverpool UK; ^3^ Care Policy and Evaluation Centre London School of Economics and Political Science London UK; ^4^ Population Health Sciences University of Bristol Bristol UK; ^5^ Centre for Health Services Studies University of Kent Canterbury UK; ^6^ Personal Social Service Research Unit, School of Social Policy University of Kent Canterbury UK

**Keywords:** advance care planning, care home, care planning, Delphi survey, nursing home, older adults

## Abstract

**Background:**

Older adult care homes in England must develop care plans on behalf of their residents and make them available to care providers. There is currently a lack of formal agreement around the key principles that should inform the care planning process.

**Objective:**

The study aimed to develop a set of key principles for care planning in older adult care homes in England.

**Methods:**

We developed 78 evidence‐based items and presented them to a panel of health and social care professionals with experience of care planning. We used two online rounds of Delphi to generate consensus (≥ 75%) on items to include in a set of key principles for care planning.

**Results:**

A set of key principles, comprising 81 items, were developed. One‐hundred participants completed Round 1, and 80 participated in Round 2. Three percent (*n* = 4/78) of the Round 1 statements did not reach agreement. Revisions primarily related to the terminology used, clarification of language and an increased emphasis on care home residents' consent and autonomy. Agreement was achieved on all statements (*n* = 78/78) in Round 2.

**Conclusion:**

Substantial agreement was achieved regarding the document's content. Future research should (a) look to develop a resource for the family and friends of care home residents to enhance their participation in care planning and (b) explore how these principles can be put into practice.

**Reporting Method:**

Study reporting was guided by the Conducting and REporting of DElphi Studies (CREDES) framework.

**Patient or Public Contribution:**

Two public involvement advisers with lived experience of caring for a relative living in a care home worked with researchers to develop the key principles and Delphi survey, recruit panel members, interpret the results from the two rounds and assist with revising the items.

## 1 Introduction

More than 250,000 people aged over 65 live in older adult care homes in England [1]. These homes provide care and support to people with daily activities such as eating, washing, dressing and socialising. In recent decades, care homes have supported people with more complex needs [2]. To meet these needs, English care homes must assess residents' needs and develop individual care plans. The Care Quality Commission (CQC), which regulates health and social care in England, states that care planning should concern a ‘person's whole life, including their goals, skills, abilities and how they prefer to manage their health’ [3]. Care homes must ensure that the people they support are involved in the ‘planning, management and review of their care’. Care providers must also make residents' care plans available to staff involved in their care [4].

Researchers have documented various approaches to care planning, including biographical training, quality‐of‐life toolkits and structured case conferences [5, 6, 7]. Qualitative research indicates that care planning practices can vary considerably between care home settings and that staff are often inadequately trained [8, 9]. A recent systematic scoping review also highlighted inconsistencies in care planning interventions [10]. The review examined 112 studies and identified only four care planning interventions in England that provided information resources for care home staff. None of these four studies provided resources for general care planning. Instead, they focused specifically on *advance* care planning (ACP). While care planning aims to understand a person's present circumstances and preferences, ACP, which often forms part of the wider care planning process, concerns future care and is often focused on palliative care [11]. ACP—which has been associated with benefits for care home residents, their family members and wider health and social care systems—should be seen as related but distinct from care planning more generally [12, 13].

Like the literature, government and voluntary guidance in England focus on ACP rather than broader care planning. Both National Health Service (NHS) England and the National Institute for Health and Care Excellence (NICE) have produced ACP guidance [11, 14]. Third‐sector organisations have also developed resources about ACP for care home residents and their family and friends. Marie Curie Hospice, for example, provides advice on how to make an advance care plan [15]. Alternatively, Hospice UK has developed an online tool called ‘Planning Ahead’ to help people think through their end‐of‐life care preferences, while Dementia UK has created an ACP template and accompanying guide [16, 17]. The authors are not aware of any specific guidance for English care homes about care planning more broadly.

A review of existing literature shows inconsistent care planning practices and a lack of guidance for care planning (rather than ACP) in care homes. This paper responds to these phenomena and presents findings from a two‐round modified Delphi survey to establish key principles for care planning in older adult care homes in England.

## 2 Methods

### 2.1 Study Design

**Figure 1:**
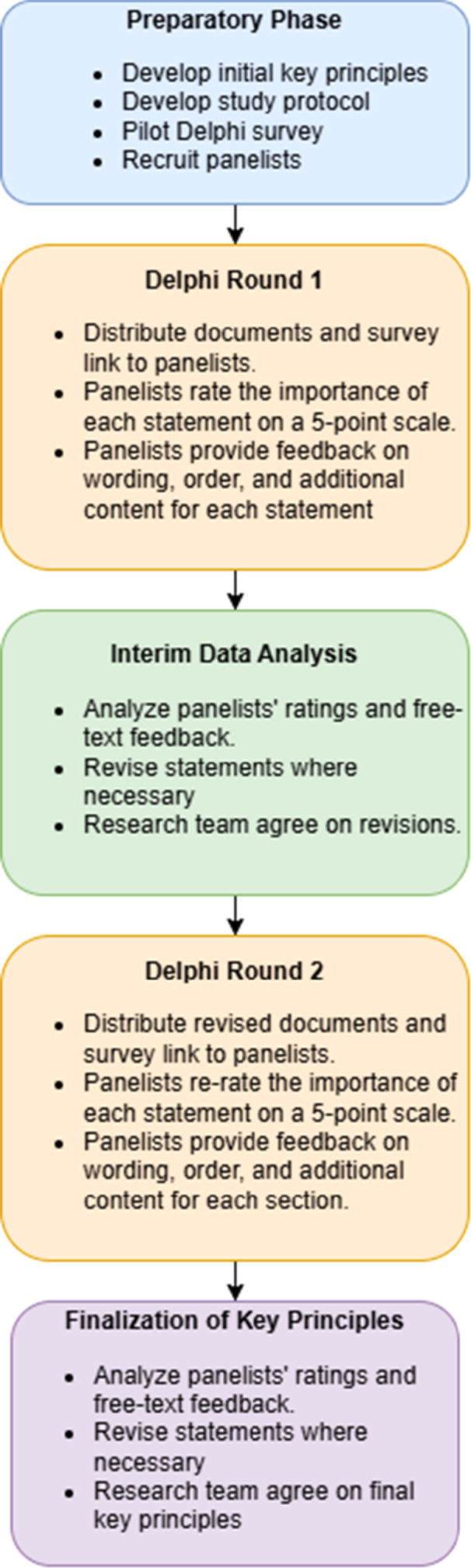
Project flow chart.

A modified Delphi survey was conducted to create a resource outlining key principles for care planning in care homes for older adults in England. This method has previously been used to develop best practice guidance, including guides relating to the care of older adults [18, 19]. The present study's methods are described in a corresponding protocol and are summarised in Figure 1 [20]. The reporting was guided by the Conducting and REporting of DElphi Studies (CREDES) framework (see Supporting Material 9 for further information) [19].

### 2.2 The Development of the Key Principles

The initial key principles document comprised seven sections (see Table 1) and was developed through synthesis of material generated at earlier phases of the project, namely: consultations with health and social care professionals involved in providing support to care home residents (including activity coordinators, general practitioners, nurses, care home managers and deputy managers) and a systematic scoping review [8, 10, 21]. Furthermore, a review of ‘grey literature’, which comprised documents and publications produced by third‐sector organisations, government bodies and research institutions, was conducted to develop a more up‐to‐date picture of care planning practices [11, 22, 23]. Key concepts, recurring themes and emergent recommendations were systematically extracted from these diverse sources. This extracted information was then grouped and categorised based on conceptual similarity. This comprehensive synthesis ensured that the initial statements were evidence‐informed, reflective of professional and organisational practices, and representative of the current understanding and identified needs related to care planning in older adult care homes.

**Table 1:** Overview of sections.

Section	Focus
1	What is the purpose of a (n advance) care plan?
2	How can care planning be approached in a person‐centred way?
3	What should be contained within a care plan?
4	When will a care plan be developed and updated?
5	Who is likely to contribute to a care plan?
6	Who should have access to a care plan?
7	Digital care plans

Two Public Involvement (PPI) advisers, who were involved in the previous phase of the project, reviewed the initial key principles document, which was amended accordingly.

### 2.3 Delphi Survey Piloting

The research team (J.T., T.C., J.D., Y.H., N.S., M.T. and M.P.) transformed the initial key principles into 78 statements. These statements were then integrated into a survey hosted on Qualtrics.

Two care home managers piloted the Round 1 survey on Qualtrics and met with two researchers (J.T. and J.D.) to provide feedback on the clarity and appropriateness of the questions and the key principles. The layout and format of the Round 1 survey and the wording of several statements were modified based on the feedback received. No additional statements were added following the pilot. The people who piloted the survey did not take part in the final modified Delphi surveys.

### 2.4 Panel Selection and Recruitment

To minimise selection bias, participants were recruited from a diverse range of organisations to reduce the risk of over‐representation from specific regions or professional backgrounds [24]. Recruitment involved purposive and convenience sampling techniques, utilising various approaches to ensure the assembly of a multidisciplinary panel. Members of the research team used the CQC's website to identify older adult care homes in their regions and invited their staff members, via email, to take part in the study. Information about the study was also shared with relevant intermediary organisations. These included care home associations, local Enabling Research in Care Homes (ENRICH) networks, the National Activity Providers Association, the National Care Forum, the British Society of Gerontology's Special Interest Group on Care Homes, trade unions and charities that advocate for care workers (see Supporting Material 1 for further information). Members of the research team also approached care homes that had taken part in previous research studies. Prospective panelists were encouraged to share the research opportunity, such as with colleagues working at different care homes, who met the eligibility criteria [25].

### 2.5 Inclusion Criteria

To become a panel member, prospective panelists were required to confirm that they were over the age of 18 and had been involved in care planning in older adult care home settings in one or more of the following ways: writing care plans, reviewing the contents of care plans, using a care plan as part of providing care and support, supervising care planning, delivering training relating to care planning, or contributing to one or more sections of a care plan. Panelists could select an ‘other’ option and provide more details. Responses were screened for eligibility by a member of the research team.

Only eligible panelists who completed the first survey were invited to complete the second survey.

### 2.6 Ethics and Consent

This study received ethical approval from the University of Kent's Division for the Study of Law, Society and Social Justice Research Committee Ethics Panel (reference: 1006) on 9 April 2024. Before participation, all individuals were provided with a participant information sheet detailing the study's purpose, procedures, potential risks and benefits. Participants were also provided with a briefing paper which explained how the statements had been developed and what a Delphi survey involves. Informed consent was obtained from all participants before completing each Delphi survey.

### 2.7 Data Collection

Data collection took place online between 21 June 2024 and 1 October 2024. Participants were invited to submit feedback over two rounds. The use of two rounds is consistent with previous Delphi projects in care home settings [18, 26]. We anticipated that two rounds would provide sufficient opportunity for participants to offer comprehensive feedback and for effective consensus to be reached, while also balancing participant burden and ensuring study feasibility. Data collection followed the following sequential stages [27].

### 2.8 Delphi Round 1

In line with previous modified Delphi studies, Round 1 panelists received the draft key principles document (Supporting Material 2), a briefing on its development, an online survey link and a participant information sheet [18, 28, 29, 30]. Panelists were asked to consult the key principles document when completing the survey questions.

Round 1 panelists were asked to rate the importance of each of the statements on a five‐point unipolar scale as follows: ‘1 = Not at all important’, ‘2 = Slightly important’, ‘3 = Somewhat important’, ‘4 = Very important’ and ‘5 = Extremely important’. Panelists could also select a ‘I don't know’ option. The wording was informed by previous Delphi studies [31, 32, 33].

For each statement, panelists were invited to suggest revisions to the wording of the statements, comment on the order of the statements, suggest additional content and provide further comments. Panelists could also make additional comments towards the end of survey.

The Round 1 survey included questions about panelists' professional backgrounds, such as job title(s) and time spent working within the care home sector, as well as demographic details.

In England, the CQC is responsible for inspecting care homes under the Health and Social Care Act 2008. Following an inspection, a care home is given one of four ratings: ‘Outstanding’, ‘Good’, ‘Requires Improvement’ or ‘Inadequate’. Round 1 panelists who worked for a single care home were asked to indicate the care home's current CQC rating.

The Round 1 survey was open from 21 June until 1 August 2024.

### 2.9 Delphi Round 2

Round 2 panelists were sent (a) a revised set of key principles (Supporting Material 3), (b) an explanation of the changes that had been made (Supporting Material 4), (c) a participant information sheet, (d) details of their scores for each statement compared with the distribution of all scores (Supporting Material 5), and (e) a link to the online survey. Panelists' comments included as part of the explanation of changes were anonymised to limit authority bias [34].

The same 5‐point unipolar scale was used as the first survey and, owing to the number of revisions made after Round 1, Round 2 panelists could provide feedback at the end of each section. Round 2 panelists were asked to indicate their job title(s) from a list of options based on the free‐text responses received at Round 1 and, if applicable, the name of the local authority in which their care home(s) was located. The Round 2 survey was open from 6 September until 1 October 2024.

### 2.10 Data Analysis and Definition of Consensus

There is no agreed‐upon definition for what constitutes consensus within a Delphi study. Previous studies have defined a consensus as being between 51% and 80% agreement [35]. This study defined consensus as being when ≥ 75% of panel members rated a statement as ‘very important’ or ‘extremely important’ on the five‐point unipolar scale. This threshold is consistent with previous research studies [35].

The study protocol [20] stated that only statements whose ratings did not meet the consensus threshold—≥ 75% of panelists rated the statement in the lower two categories (‘Not at all important’ and ‘Slightly important’) or in the higher two categories (‘Very important’ and ‘Extremely important’)—would be revised in light of panelists' responses. However, respondents' free‐text responses indicated that some statements rated as ‘Very important’ or ‘Extremely important’, and exceeded the consensus threshold, merited revisions.

Following the first round, two researchers (J.T. and a combination of T.C., J.D., Y.H., N.S. and M.T.) independently reviewed the panelists' free‐text feedback for each section. Each comment was coded as either (a) ‘agrees with current statement’, (b) ‘disagrees with current statement’ or (c) ‘agrees with current statement but suggests some revisions’. All revisions were agreed upon by both researchers before being shared with the wider research team, along with an explanation of the changes. Before Round 2, all changes made to the key principles document were agreed upon by all members of the research team.

Following Round 2, two researchers (J.T. and a combination of T.C., J.D., Y.H., N.S. and M.T.) independently reviewed all the free‐text feedback provided by panelists at the end of each of the sections. Panelists' comments either related to specific statements or to the section. The same process as Round 1 was followed. The whole research team met to agree upon the final set of key principles.

### 2.11 Patient and Public Involvement

Two relatives of care home residents were actively involved throughout the study. They helped to draft the initial key principles document, supported the recruitment of panel members, helped to interpret the results from the two rounds of the modified Delphi study and assisted in revising the key principles document.

## 3 Results

### 3.1 Panelists Characteristics

Round 1 was completed by 100 panelists and Round 2 by 80 panelists. Table 2 shows that 67 (83.8%) of the 80 panelists who completed both rounds identified as female and 12 (15%) as male. Most panelists described their ethnicity as White (*n* = 60, 75%) and were aged 35–64 (*n* = 55, 68.9%).

**Table 2:** Demographics of the 80 panelists who completed both survey rounds.

Panel composition	*N* (%)
Gender	
Female	67 (83.8)
Male	12 (15.0)
Prefer not to say	1 (1.3)
Ethnicity	
Asian or Asian British	4 (5.0)
Black/African/Caribbean/Black British	15 (18.8)
White	60 (75.0)
Prefer not to say	1 (1.3)
Age category	
18–34	19 (23.8)
35–64	55 (68.8)
65+	6 (7.5)

The panel comprised people with varied levels of experience. Table 3 shows that most panelists (*n* = 49, 61.3%) had worked in the older adult care home sector for over 10 years. Panelists reported being involved in care planning in a variety of roles, including as an administrator (*n* = 2, 2.5%), care assistant (*n* = 16, 20.0%), managing director (*n* = 2, 2.5%) and a chief executive (*n* = 1, 1.3%). Registered managers (*n* = 27, 33.8%) comprised the largest single role reported by panelists.

**Table 3:** Professional backgrounds of the 80 panelists who completed both survey rounds.

Panel composition	*N* (%)
Length of time spent working in the older adult care home sector	
Up to 1 year	2 (2.5)
1–2 years	4 (5.0)
2–5 years	16 (20.0)
5–10 years	9 (11.3)
More than 10 years	49 (61.3)
Roles that panelists held while being involved in care planning*	
Executive and Management^1^	45
Clinical and Healthcare Professionals^2^	25
Care and Support Staff^3^	36
Administrative and Quality Assurance^4^	7
Other^5^	7

* Panelists could select all that apply.

^1^ Managing director, chief executive, operations manager, deputy manager, general manager, registered manager and home manager.

^2^ Clinical psychologist, clinical lead, occupational therapist, deputy clinical lead, physiotherapist, registered nurse and junior sister.

^3^ Senior care assistant, care assistant, health care assistant, senior carer, carer and health care support worker.

^4^ Administrator, quality and compliance lead, and NVQ assessor/trainer.

^5^ Team leader, research and policy and turnaround managers.

Table 4 shows that panelists represented homes who had received the full range of CQC ratings. Most panelists worked in homes that had been rated ‘Good’ (*n* = 33, 41.3%) or ‘Outstanding’ (*n* = 15, 18.8%) by the CQC. Panelists were recruited from care homes across England.

**Table 4:** Details of the care homes in which the 80 panelists who completed both survey rounds worked.

Panel composition	*N* (%)
CQC rating of the home in which you work	
Outstanding	15 (18.8)
Good	33 (41.3)
Requires improvement	6 (7.5)
Inadequate	1 (1.3)
Home has not yet been rated	4 (5.0)
Not applicable—for example, I work for more than one care home/I don't work for a care home	15 (18.8)
Prefer not to say	6 (7.5)
Region in which the panelists' care homes are located^1^	
East Midlands	9 (11.3)
East of England	5 (6.3)
North East	1 (1.3)
North East and North Cumbria	1 (1.3)
North London	1 (1.3)
North West	7 (8.8)
South East Central	12 (15.0)
South East Coast	2 (2.5)
South West Central	7 (8.8)
South West Peninsula	2 (2.5)
West Midlands	9 (11.3)
Yorkshire and the Humber	2 (2.5)
East Midlands	9 (11.3)
East of England	5 (6.3)
North East	1 (1.3)
Not applicable—for example, the participant did not work for a care home	13 (16.3)
Prefer not to say	9 (11.3)

^1^ Regions align with the NIHR's Regional Research Delivery Networks.

### 3.2 Round 1

In Round 1, 74 out of 78 statements met the study's consensus threshold, with over 75% of panelists rating each statement as ‘Very important’ or ‘Extremely important’. Of the four statements that did not meet the threshold, two were revised and retained (R1:1.8 and R1:6.3) and two statements were removed (R1:4.12 and R1:7.2). Revisions were made to 73 statements that exceeded the pre‐defined consensus threshold. In light of panelists' feedback, four statements (R1.4.1‐4) that exceeded the pre‐defined consensus threshold were combined to make two statements (R2:4.1 and R2:4.2). Five additional statements were provided at the start of section three to provide a clearer definition of person‐centred care (R2:2.0). Following Round 1, the revised document comprised a total of 79 statements (Box 1).

Box 1: **Explanation of terminology**

The following shorthand is used in the commentary below:

− R1:x.x refers to the statements that were shared with the first round of panelists. The first number refers to the section and the second the statement.
− R2:x.x refers to the statements that were shared with the second round of panelists.
− Final:x.x refers to the finalised version of the statements, which were developed in light of the Round 2 feedback.
− A full list of the statements, and a full breakdown of scores, for Rounds 1 and 2 can be found in Supporting Files 6 and 7, respectively. A full list of the statements of the final document can be found in Supporting File 8.

A full breakdown of the revisions that took place after Round 1, along with examples of the feedback which informed these revisions, can be found in Supporting File 4.

### 3.3 Round 2

A total of 80 panelists completed the Round 2 survey. Due to an administrative error, one statement (R2:6.3) was omitted from the second Delphi survey. This statement was rated as ‘Very important’ or ‘Extremely important’ by 76.3% of Round 1 panelists. Based on feedback from Round 1, minor changes were made to the statement, and it was subsequently included in the final version document (Final:6.3). All statements included in the second Delphi survey achieved the study's consensus threshold.

Following an analysis of panelists' qualitative comments, minor changes were made to 24 statements and two further statements were added (Final:3.20b, 7.10). The final document comprised 81 statements.

### 3.4 Overview of Respondents' Scores by Section

**Figure 2:**
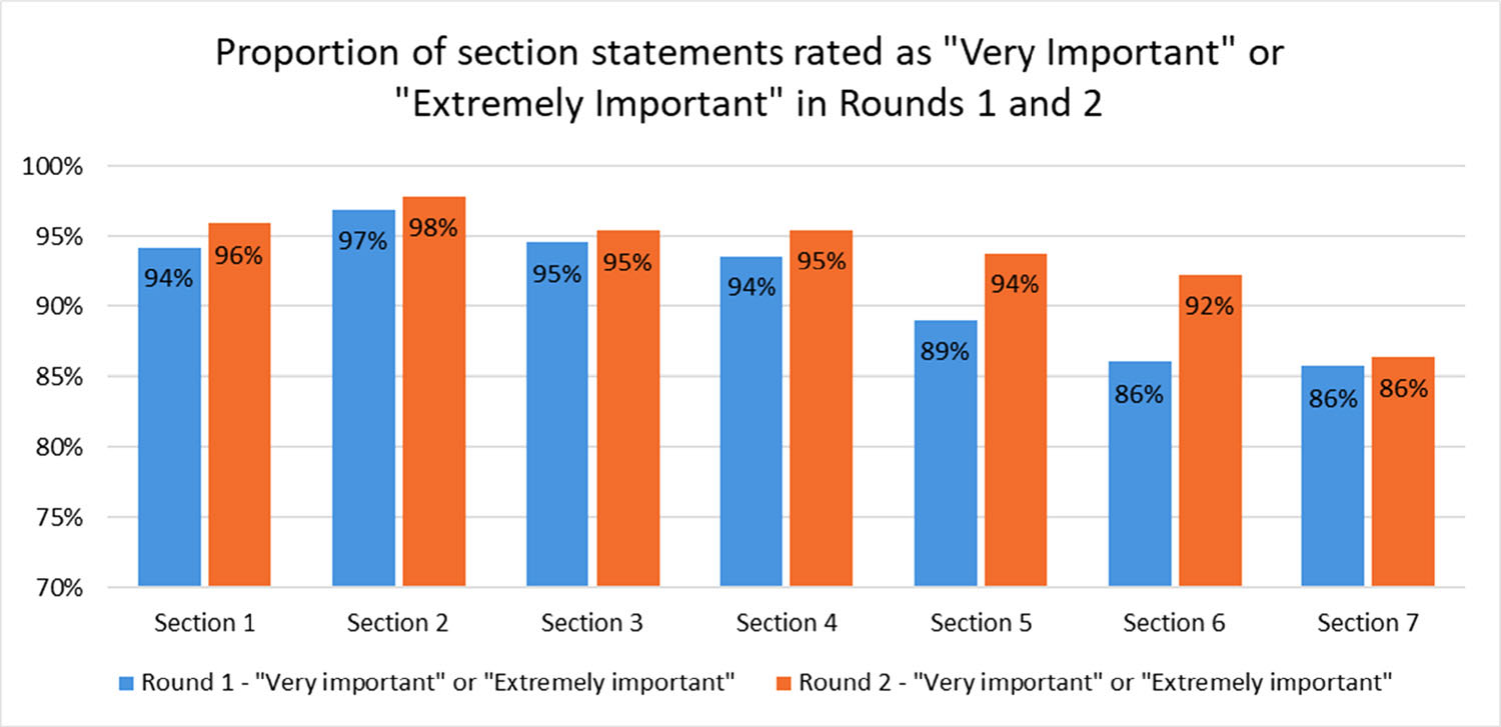
Summary of section changes.

Figure 2 shows that the proportion of participants who rated the statements as either ‘Very important’ or ‘Extremely important’ rose for all seven sections between Rounds 1 and 2.

### 3.5 Summary of Changes

#### 3.5.1 Section 1: What Is the Purpose of a(n Advance) Care Plan?

This section defines the purpose of care planning. Terminology was updated from ‘resident’ to ‘person’ to emphasise person‐centred care, and ‘quality‐of‐care standards’ was changed to ‘relevant legislation, CQC and NICE guidelines’ for greater specificity (Final:1.9).

#### 3.5.2 Section 2: How Can Care Planning Be Approached in a Person‐Centred Way?

This section addresses a person‐centred approach to care planning. Following feedback, five new statements were added at the beginning of the section (R2:2.0). These described the key features of person‐centred care and what this can help to achieve. All five were highly rated (at least 95% ‘very important’ or ‘extremely important’) in Round 2.

#### 3.5.3 Section 3: What Should Be Contained Within a Care Plan?

This section outlines information to include within a care plan. Following panelists' feedback, the document was revised to emphasise the importance of emotional well‐being (Final:3.13) and documenting residents' specific preferences for support (Final:3.14). More details were provided about the types of information to include in an advance care plan (Final:3.16‐22).

#### 3.5.4 Section 4: When Will a Care Plan Be Developed and Updated?

This section addresses care plan development and revision. It was revised to emphasise collecting pre‐arrival information and updating care plans once a person is settled. A statement suggesting 6‐monthly reviews (R1:4.12) was removed after Round 1 due to low support for specific review frequencies.

#### 3.5.5 Section 5: Who Should Contribute to a Care Plan?

This section outlines who should contribute to care plans. Panelists suggested that managers or senior care staff should produce plans with input from the wider care team and external professionals (Final:5.3). Revisions also clarified that a person's involvement is informed by their capacity (Final:5.1).

#### 3.5.6 Section 6: Who Should Have Access to a Care Plan?

This section addresses care plan access. Revisions clarified that a person's attorney should only be able to view information for which they have authority (financial or health and welfare) (Final:6.2). Panelists strongly supported access for care home staff, including agency staff, and external professionals. This led to revisions emphasising data protection (Final:6.4‐5).

#### 3.5.7 Section 7: Future Directions for Care Planning

This section on future care planning directions received the lowest importance ratings (85.6% Round 1 and 86.4% Round 2). Feedback led to the removal of one statement on staff engagement (R1:7.2) and slight moderation of language in others. For example, the statement ‘digital care plans can help to reduce the amount of time to complete and review care plans’ (R2:7.1) became ‘digital care plans can, over time, help to reduce the amount of time to complete and review care plans’ (Final:7.1).

Despite this, panelists highlighted the growing importance of digital care planning, emphasising staff training and costs. In response to panelists' feedback, an additional statement was added to encourage care homes to ensure that the digital care platforms do not inadvertently exclude residents and important people in their lives from contributing to the care planning process (Final:7.10).

## 4 Discussion

Principles to inform care home practices are most likely to be adopted when they have been informed by those involved in applying them to their work [18]. With this in mind, we conducted a modified Delphi study to develop a set of key principles for care planning in older adult care homes. This is the first study, to our knowledge, which has used this approach to create a resource to inform care planning practices in older adult care homes in England.

The discussion explores three cross‐cutting features of the guide—promoting autonomy, strengthening relationships and responsive care planning—and how they relate to existing research, practice and opportunities for future learning.

### 4.1 Promoting Autonomy

Many of the statements contained within the key principles document highlight opportunities to recognise, accommodate and promote people's individual preferences. For instance, the first section notes that ‘when done well, care plans will empower a person to have as much choice, control and independence over their daily life as possible’ (Final:1.3). Likewise, Section 3 suggests that care plans should document a person's capabilities as well as how they would like to be supported (Final:3.14) and further encourages care homes to record key dates and life events to enable a person to celebrate these milestones (Final:3.5). Section 5 further suggests that a person must be involved in developing their care plan and notes that what this involvement looks like will be informed by a person's individual circumstances (Final:5.1). The importance of respecting residents' autonomy has also been emphasised in a recent ‘Practice Pointer’ article by Hyson and Fritz [36]. Fleuren et al. also view respecting individuals' autonomy as a key underlying goal of ACP [37].

While the key principles stress the importance of involving care home residents, research suggests that residents are often not consulted in practice. Smith et al. found that residents perceived as lacking capacity were frequently excluded from the care‐planning process, while even those deemed to have capacity were often excluded from discussions on sensitive topics, such as end‐of‐life care preferences [8]. Damant et al. found that adverse staffing conditions, which can lead to staff being overworked, can inhibit person‐centred care planning (PCCP) practices, which promote respecting people's autonomy and individual preferences [21].

### 4.2 Strengthening Relationships

The value of relationships is reflected in the key principle's assertion PCCP can help to ‘build trusting relationships between the person and their care team’ (Final:2.0). This finding echoes Stein‐Parbury et al.'s assertions that person centred care ‘is more than simply individualizing care’ and involves a ‘dynamic social interplay’ so that care home staff understand a resident's feelings and sense of self [38]. Likewise, Fleuren et al. consider strengthening relationships as a key part of ACP [18].

In addition to their relationships with residents, the key principles document emphasises the importance of care homes encouraging family and friends, as well as external health and social care professionals, to contribute to care planning. Indeed, residents' family and friends often continue to provide care and support once they have moved into a residential care setting [39, 40, 41]. The key principles document encourages care homes to invite family and friends and external professionals to contribute to a person's care plan (Final:4.3, 5.2) and to provide them with access to the care plan when appropriate (Final:6.3, 6.5).

Evidence suggests that family and friends value the chance to be involved in supporting their relatives' care. Bavelaar et al. and Brazil et al. found that families involved in the care planning process reported less decision‐making uncertainty and a more positive opinion of the care provided to their relative [41, 42]. Research also indicates that allowing multiple professionals to have access to a person's care plan can help improve care quality. McSweeney et al. found that multidisciplinary consultations were significantly more effective than standard practice when caring for older adults [43].

Research shows that building meaningful relationships with family, friends and external professionals is often challenging. A registered nurse, interviewed as part of Smith et al.'s study, noted that families were involved in fewer than 10% of care plans in their experience [8]. Ethnographic researchers have observed that multi‐professional and relatives' involvement in ACP can be disjointed, leading to uncertainty over who is responsible for the care plan [44]. Indeed, a review of care planning practices among community‐based older adults also found that health and social care practitioners were often uncertain about who was responsible for aspects of ACP [45]. Further research is needed to identify how best to ensure that staff and external contributors have a clear understanding of how and when they can contribute to the care planning process.

### 4.3 Responsive Care Planning

The document emphasises that care planning should be a dynamic process. Section 4, for example, recommends that ‘an effective care plan will be regularly reviewed to ensure the document reflects a person's current needs, interests, and preferences’ (Final4:5). The document further suggests that care plans should be updated to reflect changes in a person's health, well‐being, preferences, hospital admissions or new safety risks (Final4:6). Section 3 of the document also recommends that staff should record ‘when the plan was created, revised and will next be reviewed’ (Final:3.2). This approach is consistent with NHS England's recommendation that a care plan should be a ‘living’ document that will involve ‘ongoing dialogue with a person and those close to them about how to meet their current needs and those that can be anticipated in the future’ [46, 47]. Likewise, Ajibade has suggested that care planning should be viewed as a dynamic process that includes assessing needs, identifying problems, setting goals, developing interventions and evaluating outcomes [48].

Panelist strongly agreed that an ‘effective care plan’ should be ‘routinely updated’. More than 96% of respondents rated this statement as either ‘very important’ or ‘extremely important’ in Round 1 (R1:4.8). When asked when regular reviews should take place, however, panelists were divided. Suggestions ranged from once every week to once every 8 weeks. For this reason, the final document did not recommend a set period within which to review a care plan.

Smith et al. also found that the timing of reviews varied across care homes. Current guidance also varies [8]. The NICE, for example, recommends that an older person's health and social care plan should be reviewed at least once a year [49]. In contrast, AgeUK, a charity dedicated to supporting older people, recommends that local authorities should review service users' care plans within 6–8 weeks [50]. In future, policymakers and regulators, such as the CQC, may wish to provide care homes with clearer guidance on how frequently care homes should review their residents' care plans.

### 4.4 Future Research

Our Delphi panelists suggested that the family and friends of care home residents can play an important role in care planning. Future research should look to develop a resource to help inform residents' family and friends about care planning and encourage them to contribute their insights into the process. The final set of key principles comprises six pages of A4. The length of this document may restrict its use by care home staff, many of whom are working in homes that are short‐staffed [51, 52]. Future research should pilot the key principles in care homes to identify implementation barriers and assess the value of a condensed version.

While this study specifically addresses the need for care planning principles within older adult care homes in England, aligning with existing English regulatory frameworks and guidance from bodies like the CQC, NHS England and NICE, the core tenets of the developed key principles may hold broader international relevance. Indeed, PCCP has been internationally promoted across a range of health and social care settings [53, 54, 55]. The identified cross‐cutting features of these principles—promoting autonomy, strengthening relationships and fostering responsive care planning—will be fundamental to person‐centred care worldwide. Therefore, future research could also investigate the translatability and adaptability of these principles to care home settings in other countries. Such studies would need to consider how these principles interact with diverse national policies, regulatory environments and cultural contexts to ensure their effective implementation.

### 4.5 Strengths and Limitations

One of this study's strengths has been the involvement of two relatives of care home residents whose experiences directly informed the original key principles, helped interpret panelists' feedback and assisted in revising the key principles document. A second strength has been the ability to recruit a panel of experts from across England, with diverse lengths of service and a wide variety of experiences. This study also succeeded in retaining a substantial proportion (80%) of those involved in the first round. The formation of a group with such a range of professional experiences has helped to increase the generalisability and creditability of the results. The high retention rate helped to reduce attribution bias between Rounds 1 and 2.

One study limitation is that most panelists worked in care homes with ‘Good’ or ‘Outstanding’ CQC ratings. Amongst panelists who detailed the CQC rating of the home in which they worked, over 25% of Round 2 panelists reported working in a care home rated ‘Outstanding’ compared to fewer than 5% of all care homes in England [56]. This is a limitation because it may introduce a bias in the findings, as the experiences and perspectives of staff working in lower‐rated care homes are under‐represented. Future research on care planning should seek to represent the diversity of CQC ratings.

A further weakness is that the Round 1 survey was informed by findings of earlier work, so panelists were limited in the extent to which they could define the initial scope of the key principles. That said, Round 1 panelists were able to suggest revisions at the statement level, and significant changes to the key principles document followed panelists' feedback. The use of free‐text responses also enabled panelists to provide additional context to their ratings and to suggest content they felt was missing.

## 5 Conclusion

Through a modified Delphi process, we developed a set of principles to guide care home practitioners when developing person‐centred care plans for older adults living in care homes in England. Care home professionals are encouraged to promote residents' autonomy by documenting preferences and capabilities, strengthen relationships through active involvement of families and professionals, and advocate for responsive, regularly updated care plans. If implemented, this document may help to improve the consistency with which PCCP is delivered.

## Author Contributions

**Jonathan Taylor:** conceptualisation, data curation, formal analysis, writing – original draft, methodology, visualisation, investigation, project administration. **Thais Caprioli:** data curation, visualisation, writing – review and editing, methodology, investigation, formal analysis. **Jacqueline Damant:** data curation, formal analysis, writing – review and editing, methodology, investigation. **Yuri Hamashima:** data curation, formal analysis, writing – review and editing, investigation, methodology. **Nick Smith:** data curation, formal analysis, methodology, investigation, writing – review and editing, funding acquisition. **Madalina Toma:** formal analysis, methodology, investigation, writing – review and editing. **Michele Peters:** writing – review and editing, supervision.

## Ethics Statement

This project has been reviewed by and received ethics clearance from the Division for the Study of Law, Society, and Social Justice Research Committee Ethical Panel at the University of Kent (Ethics reference: 1006, approved on 9 April 2024).

## Conflicts of Interest

The authors declare no conflicts of interest.

## Supporting information

SI1 Intermediary‐organizations‐contacted.

SI2 Key‐principles‐document‐shared‐with‐round‐panelists.

SI3 Revised‐set‐of‐key‐principles‐sent‐to‐round‐panelists.

SI4‐Explanation‐of‐the‐changes‐that‐had‐been‐made‐between‐rounds‐1‐and‐2.

SI5‐Example‐of‐a‐panelists‐scores‐for‐each‐statement‐compared‐scores.

SI6 List‐of‐round‐1‐statements‐summary‐of‐respondents‐unipolar‐scores.

SI7‐List‐of‐round‐2‐statements‐and‐summary‐of‐respondents‐unipolar‐scores.

SI8‐List‐of‐final‐statements‐REVISED.

SI9‐Guidance‐on‐Conducting‐Reporting‐Delphi‐Studies‐CREDES‐Checklist.

## Data Availability

The data that support the findings of this study are openly available in ReShare at https://reshare.ukdataservice.ac.uk/. This dataset has been saved with the following title: ‘Developing a set of key principles for care planning within older adult care homes’.
