# Supplementary material for: Developing a Set of Key Principles for Care Planning Within Older Adult Care Homes: A Modified Delphi Survey
Source: Health Expect. 2025 Sep 29;28(5):e70433. doi: 10.1111/hex.70433 (PMC12477624; doi:10.1111/hex.70433)
Supplement: Supplementary file 1 — SI1 Intermediary‐organizations‐contacted. [file HEX-28-e70433-s002.docx]

# Supplementary information 1: Intermediary organizations contacted

1. Care England
2. GMB Union
3. Join Dementia Research
4. National Association of Activity Providers
5. National Care Forum
6. Skills for Care
7. The British Society of Gerontology’s Special Interest Group on Care Homes
8. The Care Workers Charity
9. The Care Workers Union
10. The National Activity Providers Association
11. The National Care Forum
12. The Outstanding Society
13. Unison
14. Unite the Union
15. Regional Care Associations, specifically:

- Bedfordshire Care Group
- Berkshire Care Association
- Buckinghamshire Care Providers Association
- Cambridgeshire Care Providers Alliance
- Care and Support West
- Care Association for Brighton & Hove and East Sussex
- Devon Care Homes Collaborative
- Dorset Care Association
- East Midlands Care Association (EMCARE)
- Essex Care Association
- Gloucestershire Care Providers Association
- Hampshire Care Association
- Independent Care Group
- Kent Integrated Care Alliance
- Leeds Care Association
- Lincolnshire Care Association
- London Care and Support Forum
- Norfolk Care Association
- Oxford Association of Care Providers
- Partners in Care
- Registered Care Providers Association
- South East Social Care Alliance
- Staffordshire Care Association
- Surrey Care Association
- West Midlands Care Association
- West Sussex Partners in Care

1. The NIHR’s Enabling Research in Care Homes (ENRICH) Networks based in the following regions:

- East Midlands
- Eastern
- Greater Manchester
- Kent, Surrey and Sussex
- North East and North Cumbria
- North Thames
- North West Coast
- North West London
- South London
- South West Peninsula
- Thames Valley & South Midlands
- Wales
- Wessex
- West Midlands
- West of England
- Yorkshire and Humber
