# Supplementary material for: Developing a Set of Key Principles for Care Planning Within Older Adult Care Homes: A Modified Delphi Survey
Source: Health Expect. 2025 Sep 29;28(5):e70433. doi: 10.1111/hex.70433 (PMC12477624; doi:10.1111/hex.70433)
Supplement: Supplementary file 2 — SI2 Key‐principles‐document‐shared‐with‐round‐panelists. [file HEX-28-e70433-s001.pdf]

Key Principles for Care Planning

1. What is the purpose of a(n advanced) care plan?

An effective **care plan** provides a snapshot of a resident's whole life, including their goals, skills, abilities and how they would like to manage their health and wellbeing. A strong care plan will acknowledge that a person's needs and interests can change over time, sometimes in response to changes to their health. When done well, care plans will empower residents to have as much independence and control over their daily life as possible.

The information contained within a strong care plan should help to:

- Identify residents' preferences and wishes each time staff provide care or support
- Identify the views of residents or their family and friends, where possible, regarding the care and support they receive
- Maintain continuity of care among external partners and collaborators
- Assess resident's health and wellbeing over time
- Assist in managing staffing levels and resources
- Demonstrate that the identified care needs comply with quality-of-care standards
- Set out what the resident's best life in the home would look like

An effective **advance care plan** will enable a care home resident to set out their preferences and priorities for future care. Advance care planning (ACP) is designed to help ensure that the care that people receive in the future is consistent with their values, goals and preferences. If not already in place, ACP can lead to the appointment of a health and welfare Lasting Power of Attorney who is legally empowered to make decisions about the treatment a resident would receive if they no longer had the mental capacity to consent.

Advanced care plans often include information about a resident's **end of life care** including where the resident would like to die, if the resident has completed a "do not attempt cardiopulmonary resuscitation" (DNACPR) form, and any, religious and/or spiritual requests. Advanced care plans may also document a resident's future treatment preferences and where they would like to spend their last days.

2. How can care planning be approached in a person-centred way?

A **person-centred care** plan will help to meet a person's needs and preferences. A person-centred care plan has the following qualities:

- It provides a holistic understanding of a resident as an individual, including their history, current interests and future ambitions. It will detail:
  - The social, emotional, and health issues for which a resident requires support
  - The resident’s personal values and priorities for their care
  - The resident’s capabilities as well their needs
- It engages the resident, and key stakeholders, in decision-making. This can be achieved by:
  - Inviting residents to take the lead in discussing the care plan’s contents, wherever possible
  - Taking reasonable steps to meet residents’ communication (e.g., plain English, information available in Braille, translators) and sensory needs (e.g., hearing aids, glasses).
  - Including input from important people in their life
  - Ensuring that residents are aware of all the available options and provide them with the information necessary to make informed decisions. With residents’ consent*, family and friends can also be provided with this information to help them assist the resident in a making a decision.
    * This consent may be delegated to a person(s) granted Lasting Power of Attorney.
  - Including input from external care providers, professionals and organisations involved in promoting the resident’s health and wellbeing

3 . What should be contained within a care plan?
Care plans will contain different sections. High quality care plans are likely to include:

1. A recent photograph of the resident
2. Details about the **care plan itself:**
    - A record of when the plan has been created, reviewed, updated and modified and when the care plan will next be reviewed
3. Background information about the **resident's history**, including details of:
    - The resident's life immediately prior to moving into the care home
    - The resident's family, culture and religion
    - Key dates and life events, such as significant holidays, anniversaries, and service honours
4. Information about a resident's **hobbies, interests and aspirations, past and present**:
    - Information about how to support the resident's current goals
    - Information about activities the resident would/would not like to take part in and environments that they feel/do not feel comfortable in
5. Information about the **risks** that the resident may face, and **steps that can be taken to mitigate them** in a person centred way
6. Information about **forthcoming appointments** and details of who will be responsible for arranging transportation and accompanying the resident, these could be medical or social appointments
7. Information about the **resident's health**, including, but not limited to:
    - Vital signs
    - Medication
    - Nutrition and hydration needs
    - History of physical and mental health
8. Information about the resident's **day-to-day care needs** and **preferences**, including:
    - The resident's capability to meet their own day-to-day needs and any preferences for receiving support
    - Details of any specialist equipment that the resident may need, such as adapted cutlery or hearing and mobility aids
9. Information about a resident's **end of life care**, including:
    - Where the resident would like to be cared for
    - Details of religious, spiritual and/or cultural practices
    - Key people to involve
    - Who the resident would like to be with them in their final moments
    - Palliative medical care and resuscitation preferences
    - Funeral arrangements
    - Whether arrangements have been made for organ or body donations

4. When will a care plan be developed and updated?

A well-developed care plan will provide an accurate and up-to-date account of a resident's needs and interests. Care plans should be thought of as a "live" document that will be continually updated. With this in mind, there are three key time points at which care plans are likely to be developed and revised:

1. Prior to, or shortly after, a resident begins residence at a care home.
   - Where possible, key information about a resident (such as their health conditions, significant bereavements, important people in their life, the equipment that they use, and their medical needs) should be included in a care plan prior to them moving into a care home. This information – which could be gathered as part of a pre-admission assessment - may be obtained by talking to the resident, their General Practitioners (GP) or social worker and, with the resident's consent, their family and friends.
   - In the first 2-4 weeks following a resident's arrival at a care home, as staff begin to get to know the resident better, it is often helpful to set aside time to develop a care plan.
2. A care plan should also be updated in response to significant changes or incidents in a resident's life, such as a fall, the development of new friendships, personal achievements, new hobbies and interests, a change in their health, or a hospital admission.
3. Thereafter, an effective care plan will be routinely updated, possibly in the form of regular and more meaningful and holistic reviews, to ensure the document reflects a resident's current needs and interests. Where possible, and with a resident's permission, family members may contribute to these reviews.
   - To ensure that care plans remain accurate and up-to-date, regular reviews are likely to take place at least every 6 weeks. These reviews can provide an opportunity to assess the contents of a resident's care plan and discuss whether any changes need to be made
   - More detailed care plan reviews may take place every six months

5. Who is likely to contribute to a care plan?

- Where possible, residents should be involved in developing and reviewing their care plans
- Were possible, with a resident's agreement, involve their family and friends as they can often provide valuable information
- Senior care or nursing staff are usually responsible for writing care plans; however, valuable information can also be provided by front line care workers and non-care staff - such as members of the housekeeping and catering teams.
- External health and care professionals, such as medical consultants, social workers, GPs, and occupational therapists, may contribute to specific parts of the care plan.

6. Who should have access to a care plan?

To be most useful, care plans will need to be accessible to:

- The residents themselves
- People who have legal power of attorney for the resident
- Members of a resident's 'circle of care', such as named family and friends, that the resident or their LPA has consented to see their care plan/ certain sections of the care plan
- Care home staff, including bank and agency staff
- External health and care professionals, such as social workers, GPs, and pharmacists

7. Future developments in care planning

Technology, such as digital care planning software, is playing an increasingly important role in supporting care planning. Digital care plans can:

- Help to reduce the amount of time to complete care plans
- Improve staff engagement in care planning
- Produce aggregate data which can help the home plan for the future
- Allow information to be securely and quickly shared with relevant stakeholders, such as health and social care professionals and a resident's family and/or friends

Care homes that are interested in adopting digital care plans may need to consider:

- Whether they have sufficient internet coverage across their site(s)
- The associated costs of software licences/updates, devices, network and data security, support and maintenance
- If the software selected allows staff to develop person-centred care plans
- Whether the digital care plan can be made accessible to all the relevant people involved in supporting the resident, while ensuring that only appropriate people will be able to update the digital care plan
- The time commitment likely to be associated with:
  - Transitioning from paper to digital care plans
  - Training and supporting staff to use digital care planning packages as well as meeting ongoing training needs
