# Supplementary material for: Developing a Set of Key Principles for Care Planning Within Older Adult Care Homes: A Modified Delphi Survey
Source: Health Expect. 2025 Sep 29;28(5):e70433. doi: 10.1111/hex.70433 (PMC12477624; doi:10.1111/hex.70433)
Supplement: Supplementary file 4 — SI4‐Explanation‐of‐the‐changes‐that‐had‐been‐made‐between‐rounds‐1‐and‐2. [file HEX-28-e70433-s005.pdf]

Original	Revised	Examples of feedback provided
**Section 1: What is the purpose of a(n advanced) care plan?**		
1.1 An effective care plan provides a snapshot of a resident's whole life, including their goals, skills, abilities and how they would like to manage their health and wellbeing.	1.1 An effective care plan provides a summary of a person's life, including their goals, skills, abilities and the support they need to manage their health and wellbeing.	"I think an effective care plan provides more than a snapshot of a residents whole life, the word snapshot doesnt feel right"<br><br>"I don't think snapshot is the right word, I'd say a short summary of the important parts of a resident's life."
1.2 A strong care plan will acknowledge that a person's needs and interests can change over time, sometimes in response to changes to their health.	1.2 A meaningful care plan will acknowledge that a person's abilities, needs, interests and preferences, can change over time.	"'A strong care plan' sounds impersonal ... From a resident or person-centred perspective, it would be 'A meaningful care plan'".<br><br>"This does not only reflect on their health but also preferences as we have taste changes and interest changes as we age and develop"
1.3 When done well, care plans will empower resident to have as much control and independence over their daily life as possible.	1.3 When done well, care plans will empower a person to have as much choice, control and independence over their daily life as possible.	"Choice should also be included in this assessment"
1.4 The information contained within a strong care plan should help to:<br>• Identify residents' preferences and wishes each time staff provide care or support	1.4 The information contained within a meaningful care plan should help:<br>• To identify a person's care needs, preferences and wishes each time staff provide support	"Include 'wishes and needs'"<br><br>"This should also include NEEDS as needs can be different to wishes"
1.5 The information contained within a strong care plan should help to:<br>• Identify the views of residents or their family and friends, where possible, regarding the care and support they receive	1.5 The information contained within a meaningful care plan should help:<br>• To identify the views of the person regarding the care and support they receive. It can be helpful to supplement these with the views of family and friends. Wherever possible, the views of the person receiving care should be prioritised.	"The resident's view needs to be respected over their family and friends as they can sometimes be different"<br><br>"Mainly views of resident"<br><br>'Residents' views are paramount"
1.6 The information contained within a strong care plan should help to:<br>• Maintain continuity of care among external partners and collaborators	1.6 The information contained within a meaningful care plan should help:<br>• To maintain continuity of care among other health and social care professionals – such as GPs, district nurses and physiotherapists - involved in an individual's care	"In my experience that term [i.e. external partners and collaborators] would not be understood by support workers/care workers. That term is not accessible to most of the workforce and could be seen as jargon".<br><br>"Make sure GPs [and] district nurses are included"
1.7 The information contained within a strong care plan should help to:<br>• Assess resident's health and wellbeing over time	1.7 The information contained within a meaningful care plan should help:<br>• Staff to assess and monitor a person's health and wellbeing over time	"Should reference that this is regularly reviewed also"

Original	Revised	Examples of feedback provided
1.8 The information contained within a strong care plan should help to:<br>• Assist in managing staffing levels and resources	1.8 The information contained within a meaningful care plan should help:<br>• To give a clearer indication of a person's needs. This information may help to inform the resources and staffing levels required in the care home	"I agree with resources. Not sure with staffing levels - what does this mean?"<br><br>"Rather than 'managing staffing levels' I would put 'give a clearer indication of staffing needs and need of the person being supported'."
1.9 The information contained within a strong care plan should help to:<br>• Demonstrate that the identified care needs comply with quality-of-care standards	1.9 The information contained within a meaningful care plan should help:<br>• To demonstrate that the care that a person receives complies with the relevant CQC and NICE guidelines	"What do you mean by Quality of Care standards?"<br><br>"This should be more specific. Where are the "quality-of-care standards" found? I would not know this. I would understand NICE [and] CQC guidelines"
1.10 The information contained within a strong care plan should help to:<br>• Set out what the resident's best life in the home would look like.	1.10 The information contained within a meaningful care plan should help:<br>• Staff to support a person to live a fulfilling life	"I'm not keen on the way this is written. Perhaps: 'A well-devised care plan will enable residents to live fulfilled and engaged lives whilst in care'".
1.11 An effective advanced care plan will enable a care home resident to set out their preferences and priorities for future care.	1.11 An effective advance care plan will enable a person to set out their preferences and priorities for future care, including end of life care.	"Usually deals with end of life"<br><br>"'Future care' does not cover what advanced care plan is, it is more connected to end of life"
1.12 Advanced care planning is designed to help ensure that the care that people receive in the future is consistent with their values, goals and preferences.	1.12 An advance care plan is designed to help ensure that the care that people receive in the future is consistent with their values, goals and preferences.	"'Advance care plan' [rather than] an 'advanced care plan'".
1.13 If not already in place, advanced care planning can lead to the appointment of a health and welfare Lasting Power of Attorney who is legally empowered to make decisions about the treatment a resident would receive if they no longer had the mental capacity to consent.	1.13 If not already in place, advance care planning can lead to the appointment of an attorney under the terms of a Lasting Power of Attorney or a Court of Protection Deputy who is empowered to make decisions on behalf of the person.	"[Important to mention] Court of protection deputyship"<br><br>"[Include] deputyship"
1.14 Advanced care plans often include information about a person's end of life care including where the person would like to die, if the person has completed a "do not attempt cardiopulmonary resuscitation" (DNACPR) form, and any, religious and/or spiritual requests.	1.14 Advance care plans often include information about a person's end of life care including where the person would like to die, if the person has completed a "do not attempt cardiopulmonary resuscitation" (DNACPR) or ReSPECT form, and any religious and/or spiritual requests.	"Also need reference to the RESPECT forms"<br><br>"DNAR and Respect Forms are an integral part of any advanced care planning".
1.15 Advanced care plans may also document a resident's future treatment preferences and where they would like to spend their last days.	1.15 Advance care plans may also document a person's future treatment preferences and where and how they would like to spend their last days.	"[This should include details of] how and where I want to die"
**Section 2: How can care planning be approached in a person-centred way?**		
2.0	2.0 Person centred care planning affirms who a person is, their dignity, and prioritises their individual needs and wishes over and above generic routines and institutional practices. Where appropriate, person-centred care planning may involve	It was decided to add this new content and place it at the start of section 2. This content was added considering the following feedback:<br><br>"Person centred care needs to be explained"

Original	Revised	Examples of feedback provided
	consulting with a person's attorney and other important people in their lives.<br><br>Person-centred care can:<br>• ensure that a person's voice is heard, and their wishes inform the care they receive<br>• empower a person<br>• build trusting relationships between the person and their care team	"Will help to meet a person's INDIVIDUAL needs and preferences. It means putting the person who is in receipt of care services at the centre, making them the most important part of the process, not doing things easily, or standardisation"<br><br>"Person-centered care promotes individuality and supports wishes and preferences.<br><br>"In order to effectively meet a resident's needs, staff must consider their abilities. This keeps some independence and empowerment with the resident, and provides a sense of purpose and partnership working."
2.1 A person-centred care plan will help to meet a person's needs and preferences.	2.1 A person-centred care plan will help to ensure that the person's abilities are supported, and their wishes, preferences and needs are met.	"To provide person centred care you need to know all the preferences and needs of the individual"<br><br>"A Person-centred care plan will help to ensure the residents abilities, wishes, preferences and needs are met."
2.2 It provides a holistic understanding of a resident as an individual, including their history, current interests and future ambitions. It will detail:<br>• The social, emotional and health issues for which a resident requires support	2.2 It provides a complete picture of who the person is, including their history, current interests and future ambitions. It will detail:<br>• The social, cultural, behavioural, environmental, emotional and health needs for which a person requires support	The word holistic in my experience is not language which is used by care/support staff and there is a risk this is jargon and not understood by the 'end user' i.e. the person that is implementing the plan.<br><br>"I think the word 'issues' is inappropriate"
2.3 It provides a holistic understanding of a resident as an individual, including their history, current interests and future ambitions. It will detail:<br>• The resident's personal values and priorities for their care	2.3 It provides a complete picture of who the person is, including their history, current interests and future ambitions. It will detail:<br>• The person's beliefs, values and what is important to them	"Personal values and priorities should be respected"<br><br>"We are to respect the individual's personal values and beliefs"
2.4 A person-centred care plan has the following qualities:<br>• It provides a holistic understanding of a resident as an individual, including their history, current interests and future ambitions. It will detail:<br>• The resident's capabilities as well their needs	2.4 A person-centred care plan has the following qualities:<br>• It provides a complete picture of who the person is, including their history, current interests and future ambitions. It will detail:<br>• The person's abilities, to empower their independence and foster a sense of purpose, belonging and self-esteem	"Promote independence"<br><br>"It is important for staff to be aware of a resident's ability. Often staff do not allow residents to be as independent as they can be"

Original	Revised	Examples of feedback provided
2.5 A person-centred care plan has the following qualities:<br>• It engages the resident, and key stakeholders, in decision-making. This can be achieved by:<br>• Inviting residents to take the lead in discussing the care plan's contents, wherever possible	2.5 A person-centred care plan has the following qualities:<br>• It engages the person in decision-making. This can be achieved by:<br>• Inviting the person to describe the support that they would like to receive so they are central to their care choices	"'Key stakeholders' is management speak and not really directed at the team who will be delivering care"<br><br>"[Add] 'So the person is at the centre of their care choice'".<br><br>"I think inviting them to take the lead may not be very effective - they may not be aware of all options"
2.6 A person-centred care plan has the following qualities:<br>• It engages the resident, and key stakeholders, in decision-making. This can be achieved by:<br>• Taking reasonable steps to meet resident's communication (e.g., plain English, information available in Braille, translators) and sensory needs (e.g., hearing aids, glasses).	2.6 A person-centred care plan has the following qualities:<br>• It engages the person in decision-making. This can be achieved by:<br>• Taking reasonable steps to communicate in a way that the person can understand. This may involve using specialised visual, language, auditory, sensory tools - such as Braille and translators - as well as staff insights into a person's specific communication methods	"Translators when necessary".<br><br>"If a care worker finds a method which they find the resident really likes it should be documented and shared in handovers"
2.7 A person-centred care plan has the following qualities:<br>• It engages the resident, and key stakeholders, in decision-making. This can be achieved by:<br>• Including input from important people in the resident's life	2.7 A person-centred care plan has the following qualities:<br>• It engages the person in decision-making. This can be achieved by:<br>• Inviting, where appropriate, the person, or their attorney, to consent to important people in their life - who know them well and understand their individual needs and wishes and will act in their best interests - providing additional information	"Upon consent from the resident"<br><br>"If the resident agrees (capacity) or is in the resident's best interests (no capacity)"<br><br>"Just because they are a relation shouldn't give them immediate rights to be involved in their care. This should be discussed with the residents who they want involved and to what extent".
2.8 A person-centred care plan has the following qualities:<br>• It engages the resident, and key stakeholders, in decision-making. This can be achieved by:<br>• Ensuring that with resident's consent*, family and friends can also be provided with the information necessary to make informed decisions to help them assist the resident in a making a decision.	2.8 A person-centred care plan has the following qualities:<br>• It engages the person in decision-making. This can be achieved by:<br>• Ensuring that, with the consent of the person or their attorney, important people in their life can be provided with the information necessary to help them make decisions	"An important person in the resident's life at this stage could also be the carer that has a lot of input with the resident"<br><br>"If a resident loses the ability to advocate for themselves short or long term, it is important for an advocate, normally a relative, to step in and have an understanding of the individual care needs"

<table>
<tr><th>Original</th><th>Revised</th><th>Examples of feedback provided</th></tr>
<tr><td>* This consent may be delegated to a person granted Lasting Power of Attorney.</td><td></td><td></td></tr>
<tr><td>2.9 A person-centred care plan has the following qualities:
It engages the resident, and key stakeholders, in decision-making. This can be achieved by:
Ensuring that residents are aware of all the available options and providing them with the information necessary to make informed decisions.</td><td>2.9 A person-centred care plan has the following qualities:
It engages the person in decision-making. This can be achieved by:
Ensuring that the person is aware of all the available options and providing them with the information necessary to make informed decisions</td><td>“They may not be aware of all options, therefore what they communicate may be the only thing they think is possible or available and sometimes not in their best interest. I think they need to be asked lots of supportive questions and given many options (as much as they can take without being overwhelmed)”</td></tr>
<tr><td>2.10 A person-centred care plan has the following qualities:
It engages the resident, and key stakeholders, in decision-making. This can be achieved by:
Including input from external care providers, professionals and organisations involved in promoting the resident’s health and wellbeing</td><td>2.10 A person-centred care plan has the following qualities:
It engages the person in decision-making. This can be achieved by:
Including input from external health and social care professionals that will support the person's wellbeing, without disregarding the person’s wishes and preferences</td><td>“Support from others is welcomed but is not overcoming resident's decisions”.

“Accept input from other professionals but do not discount resident's wishes and those of confirmed legal advisors”.</td></tr>
<tr><td colspan="3">Section 3: What should be contained within a care plan?</td></tr>
<tr><td>3.1 Care plans will contain different sections. High quality care plans are likely to include:
A recent photograph of the resident</td><td>3.1 Care plans will contain different sections. High quality care plans should include:
A recent dignified photograph of the person, which will be updated regularly</td><td>“Needs to be updated and needs to be a dignified photo”

“Dignified photograph with their eyes open wherever possible”</td></tr>
<tr><td>3.2 Care plans will contain different sections. High quality care plans are likely to include:
Details about the care plan itself:
A record of when the plan has been created, reviewed, updated and modified and when the care plan will next be reviewed.</td><td>3.2 Care plans will contain different sections. High quality care plans should include:
Details about the care plan itself:
A record of when the plan was created, revised and will next be reviewed and who is responsible for providing particular care</td><td>“[Include] who is responsible for this”

“Record any changes to the plan and why”.</td></tr>
<tr><td>3.3 Care plans will contain different sections. High quality care plans are likely to include:
Background information about the resident’s history, including details of:
The resident’s life immediately prior to moving into the care home</td><td>3.3 Care plans will contain different sections. High quality care plans should include:
Background information about the person's history, including details of:
The person’s life immediately prior to moving into the care home and routines that were important to them</td><td>“For residents with dementia their recent routine is important”

“Preferred routines particularly”</td></tr>
<tr><td>3.4 Care plans will contain different sections. High quality care plans are likely to include:</td><td>3.4 Care plans will contain different sections. High quality care plans should include:</td><td>“Include gender identify, sexuality”</td></tr>
</table>

Original	Revised	Examples of feedback provided
• Background information about the resident's history, including details of:<br>◦ The resident's family, culture and religion	• Background information about the person's history, including details of:<br>◦ The person's gender identity, sexuality, family, culture and religion	
3.5 Care plans will contain different sections. High quality care plans are likely to include:<br>• Background information about the resident's history, including details of:<br>◦ Key dates and life events, such as significant holidays, anniversaries, and service honours	3.5 Care plans will contain different sections. High quality care plans should include:<br>• Background information about the person's history, including details of:<br>◦ Key dates and life events, such as significant holidays, anniversaries, volunteering and service honours and the support required from the home to enable the person to celebrate these milestones	"and support required from the home to enable the individual to maintain these"<br><br>"Recognising anniversaries are essential:- extra support may be required on remembering significant people in their lives"
3.6 Care plans will contain different sections. High quality care plans are likely to include:<br>• Information about a resident's hobbies, interests and aspirations, past and present:<br>◦ Information about how to support the resident's current goals	3.6 Care plans will contain different sections. High quality care plans should include:<br>• Information about a person's hobbies, interests, achievements and aspirations, past and present:<br>◦ Information about how to support the person to pursue these interests	"Current hobbies and aspirations are good to focus on"<br><br>"Achievements need to be included"
3.7 Care plans will contain different sections. High quality care plans are likely to include:<br>• Information about a resident's hobbies, interests and aspirations, past and present:<br>◦ Information about activities the resident would/would not like to take part in and environments that they feel/do not feel comfortable in	3.7 Care plans will contain different sections. High quality care plans should include:<br>• Information about a person's hobbies, interests, achievements and aspirations, past and present:<br>◦ Information about activities the person would/would not like to take part in and environments that they feel/do not feel comfortable in	"Comfortable environments are very important but residents"<br><br>"This allows staff to understand how to support a resident with their social and environmental needs, giving staff an opportunity to provide holistic care".
3.8 Care plans will contain different sections. High quality care plans are likely to include:<br>• Information about the risks that the resident may face, and steps that can be taken to mitigate them in a person centred way	3.8 Care plans will contain different sections. High quality care plans should include:<br>• Information about the key risks that the person may face, and steps that can be taken to keep them safe in the least restrictive way possible	"All risks needs to be evaluated and reduced as far as possible without restricting the resident"<br><br>"So we can try and support in areas needed and minimise risks with the least restrictive interventions"
3.9 Care plans will contain different sections. High quality care plans are likely to include:<br>• Information about forthcoming appointments and details of who will be responsible for arranging transportation and accompanying the resident, these could be medical or social appointments	3.9 Care plans will contain different sections. High quality care plans should include:<br>• Information about forthcoming appointments – such as medical or social appointments – and the support that the care home should provide to enable these to take place	"What reasonable adjustments they need to attend the appointments- this needs to be added."

Original	Revised	Examples of feedback provided
3.10 Care plans will contain different sections. High quality care plans are likely to include:<br>• Information about the resident's health, including, but not limited to:<br>• Vital signs	3.10 Care plans will contain different sections. High quality care plans should include:<br>• Information about the person's health, including, but not limited to:<br>• Vital signs, which can provide an important baseline to compare against in the future	"It's important to have a baseline at least"<br><br>"Baseline vital signs should be in the care plan".
3.11 Care plans will contain different sections. High quality care plans are likely to include:<br>• Information about the resident's health, including, but not limited to:<br>• Medication	3.11 Care plans will contain different sections. High quality care plans should include:<br>• Information about the person's health, including, but not limited to:<br>• Medication	*Apart from replacing "resident" with "person, no changes have been made to this statement*
3.12 Care plans will contain different sections. High quality care plans are likely to include:<br>• Information about the resident's health, including, but not limited to:<br>• Nutrition and hydration needs	3.12 Care plans will contain different sections. High quality care plans should include:<br>• Information about the person's health, including, but not limited to:<br>• The person's nutrition and hydration needs and preferences	"Knowing what someone likes to eat ... is extremely important"<br><br>"Nutrition and hydration is an essential part of the care plan to enable the nursing team to be aware of the likes and dislikes of resident"
3.13 Care plans will contain different sections. High quality care plans are likely to include:<br>• Information about the resident's health, including, but not limited to:<br>• History of physical and mental health	3.13 Care plans will contain different sections. High quality care plans should include:<br>• Information about the person's health, including, but not limited to:<br>• Their physical, emotional and social wellbeing	"Emotional needs should be added"<br><br>"Emotional well-being is as important"
3.14 Care plans will contain different sections. High quality care plans are likely to include:<br>• Information about the resident's day-to-day care needs and preferences, including:<br>• The resident's capability to meet their own day-to-day needs and any preferences for receiving support	3.14 Care plans will contain different sections. High quality care plans should include:<br>• Information about the person's day-to-day care needs and preferences, including:<br>• The person's capability to meet their own day-to-day needs and their preferences for how support should be delivered	"Residents should be encouraged and will work with staff to promote independence"<br><br>"It is important to know what capabilities the resident has in order to encourage them to be as independent as possible. They may have preferences on the sex of carers assisting them"
3.15 Care plans will contain different sections. High quality care plans are likely to include:<br>• Information about the resident's day-to-day care needs and preferences, including:<br>• Details of any specialist equipment that the resident may need, such as adapted cutlery or hearing and mobility aids	3.15 Care plans will contain different sections. High quality care plans should include:<br>• Information about the person's day-to-day care needs and preferences, including:<br>• Details of any equipment that the person uses, associated staff training needs, and how the equipment should be maintained	"Details of any equipment not just specialist ones."<br><br>"Yes, and how this is ... maintained. What training is needed for the staff to use the equipment?"

Original	Revised	Examples of feedback provided
3.16 Care plans will contain different sections. High quality care plans are likely to include:<br>• Information about a resident's end of life care, including:<br>• Where the resident would like to be cared for	3.16 Care plans will contain different sections. Advanced care plans, which will form part of the wider care plan, should include:<br>• Information about a person's end of life care, including:<br>• Where the person would like to be cared for	"This is important because if the resident loses capacity with old age or illness they will be unable to tell staff what they want"<br><br>"Often this is not discussed early enough and left to the last week or days of life."
3.17 Care plans will contain different sections. High quality care plans are likely to include:<br>• Information about a resident's end of life care, including:<br>• Details of religious, spiritual and/or cultural practices	3.17 Care plans will contain different sections. Advanced care plans, which will form part of the wider care plan, should include:<br>• Information about a person's end of life care, including:<br>• Details of religious, spiritual and/or cultural practices	"A must"<br><br>"Important in a multicultural society but rarely asked in my experience by younger staff who are less religious"
3.18 Care plans will contain different sections. High quality care plans are likely to include:<br>• Information about a resident's end of life care, including:<br>• Key people to involve	3.19 Care plans will contain different sections. Advanced care plans, which will form part of the wider care plan, should include:<br>• Information about a person's end of life care, including:<br>• Key people to involve	"It's important to document this".<br><br>"Very essential"
3.19 Care plans will contain different sections. High quality care plans are likely to include:<br>• Information about a resident's end of life care, including:<br>• Who the resident would like to be with them in their final moments	3.19 Care plans will contain different sections. Advanced care plans, which will form part of the wider care plan, should include:<br>• Information about a person's end of life care, including:<br>• Who the person would like to be with them in their final moments, either in-person or virtually using software such as MS Teams, WhatsApp or FaceTime	"Yes, either face to face or via tech (teams or video call). They might have family aboard, but they still can be there at the end"<br><br>"Being able to have access to this information quickly is very important"
3.20 Care plans will contain different sections. High quality care plans are likely to include:<br>• Information about a resident's end of life care, including:<br>• Palliative medical care and resuscitation preferences	3.20 Care plans will contain different sections. Advanced care plans, which will form part of the wider care plan, should include:<br>• Information about a person's end of life care, including:<br>• Palliative medical care and resuscitation preferences which may also be documented using a "ReSPECT" form	"Nearly all residents will have a 'Respect' form in place expressing their preferences"<br><br>"Respect form should be in place and completed properly"
3.21 Care plans will contain different sections. High quality care plans are likely to include:<br>• Information about a resident's end of life care, including:<br>• Funeral arrangements	3.21 Care plans will contain different sections. Advanced care plans, which will form part of the wider care plan, should include:<br>• Information about a person's end of life care, including:	"Families often don't think about this until the time"<br><br>"Knowing funeral directors to contact takes the stress off the resident's family in grief"

Original	Revised	Examples of feedback provided
	• Funeral arrangements	
3.22 Care plans will contain different sections. High quality care plans are likely to include:<br>• Information about a resident’s end of life care, including:<br>• Whether arrangements have been made for organ or body donations	3.22 Care plans will contain different sections. Advanced care plans, which will form part of the wider care plan, should include:<br>• Information about a person's end of life care, including:<br>• Whether arrangements have been made for organ or body donations	“Yep, I once had to arrange for someone's body to be donated to science”<br><br>“I did nurse one gentleman who left his brain to medical science as he had a rare form of dementia, and this was stated in his end of life wishes”
**Section 4 - When will a care plan be developed and updated?**		
4.1 A well-developed care plan will provide an accurate and up-to-date account of a resident’s needs and interests.	4.1 A care plan should be regularly reviewed, and updated, if necessary, to provide an accurate account of how to care for a person safely while meeting their needs and supporting their interests and preferences.	“As resident choice changes this needs to reflect that.”<br><br>“Ensures that caregivers has the same understanding about the safety of a resident, and that risks are managed properly and consistently”<br><br>“It is essential it is always current. It is a live document.”
4.2 Care plans should be thought of as a “live” document that will be continually updated.	[See revised statement 4.1]	
4.3 When will a care plan be developed and updated?<br>- Prior to, or shortly after, a person begins residence at a care home.	[See revised statement 4.4]	“means the admission process can be smoother and more welcoming – and that their needs and choices can be met”<br><br>“As part of the pre-admission assessment, any health and safety information should be gathered so staff can prepare appropriately for the admission”
4.4 Where possible, key information about a resident (such as their health conditions and medical needs) should be included in a care plan prior to their admission to a care home.	4.4 To provide safe and person-centred care from the outset, it is very important to gather as much information as possible prior to person’s arrival in a care home. This information should include a person’s needs, interests and preferences and can be used as the foundation for their care plan.	
4.5 This information - which could be gathered as part of a pre-admission assessment - may be obtained by talking to the resident, their General Practitioners (GP) or social worker and, with the resident’s consent, their family and friends.	4.5 The information collected prior to a person’s arrival may be obtained by talking to them and, with their consent or that of their attorney, contacting previous care settings, health and social care professionals, and their family and friends.	“Wording needs changing that with residents’ consent to be before speaking to GP and Social Worker. Also include and other health care professionals as we often speak to hospital Physio or OT or SALT etc”
4.6 In the first 2-4 weeks following a person’s arrival at a care home, as staff begin to get to know the resident better, it is often helpful to set aside time to develop a care plan.	4.6 Shortly after a person’s arrival, as staff begin to get to know them better, it is important to set aside time to update their initial care plan.	“remove “often helpful” and replace with “important””<br><br>“Should not wait 2 to 4 weeks, care plan should be written asap after admission, we for example do all within 7 days…”<br><br>“But this can take longer and new residents can take time to settle in a new environment”
4.7 A care plan should also be updated in response to significant changes or incidents in a resident’s life, such as a fall, the development of new friendships, personal	4.7 A care plan should be updated in response to changes in a person’s life. These changes could relate to their health, social or emotional wellbeing, interests and preferences, a hospital admission, the death of an important person in their life, their	“I think it should read that the care plan should be updated with any change. Otherwise it is left to the person to decide what they believe to be significant”

Original	Revised	Examples of feedback provided
achievements, new hobbies and interests, a change in their health, or a hospital admission.	goals and ambitions, abilities and newly identified safety risks. [this statement now comes after statement 4.8]	"New likes and dislikes. Falls, hospital admission and discharge, health improvement or non-improve. A lot more can be added"
4.8 Thereafter, an effective care plan will be routinely updated, possibly in the form of regular and more meaningful and holistic reviews, to ensure the document reflects a resident's current needs and interests	4.8 An effective care plan will be routinely reviewed to ensure the document reflects a person's current needs, interests, and preferences. Reviews can take two forms: (1) in response to changes and/or (2) in accordance with a prearranged timeframe.	"The statement is a bit vague. Words like meaningful and holistic are not specific and how can they be measured?"<br>"Routinely updated however should be when required, as statement above I do believe that sometimes the 4 weekly reviews are not necessary for some residents."<br><br>"We complete resident of the day monthly. Where all care plans and risk assessments are updated."
4.9 Where possible, and with a resident's permission, family members may contribute to these reviews.	4.9 Where possible, and with a person's consent or that of their attorney, significant people in their lives, such as family or friends, may contribute to prearranged reviews. [this statement now comes after statement 4.10]	"Wording permission to consent ... not all will have family members, rather significant others as identified by the resident."<br><br>"Where possible if a resident lacks capacity and has an LPOA or of they have full capacity and would like their families input (permission would need to be given)."
4.10 To ensure that care plans remain accurate and up-to-date, regular reviews are likely to take place at least every six weeks.	4.10 If no changes have been observed, prearranged reviews should take place once every four to six weeks to ensure that care plans remain accurate and up-to-date.	"In our care home, we update care plans as soon as change occurs. Routine reviewers are completed monthly."<br><br>"All care plans need to be reviewed regularly especially when there is a change in care needs, if no changes they need to be reviewed at least monthly."<br><br>"I feel that this is a poor comment, the word likely refers to something that should or could happen, and the timescale is restricting, maybe offer a ballpark time frame, 4 to 6 weeks"
4.11 Regular reviews can provide an opportunity to assess the contents of a resident's care plan and discuss whether any changes need to be made.	4.11 Prearranged reviews, which should involve the person and, if applicable, their attorney, will provide an opportunity to assess the contents of their care plan, identify any changes in their care needs and document how these will be met.	"Should say with whom it will be discussed"<br><br>"You could speak to the individual or family members and ask if they would like to add anything else"
4.12 More detailed care plan reviews may take place every six months	[Item removed]	*This statement has been removed because fewer than 75% of people who completed the survey agreed on a date for when more detailed care plan reviews should take place.*

Original	Revised	Examples of feedback provided
		People's comments also indicate that there is no consensus regarding whether there is a need for more detailed care planning reviews.
	**Section 5 - Who is likely to contribute to a care plan?**	
5.1 Where possible, residents should be involved in developing and reviewing their care plans.	5.1 A person must be involved in developing and reviewing their care plans. The manner of a person's involvement will be informed by their capacity – for example, people with limited capacity may require a proxy to assist them.	"I am a little worried about the word should, as it could mean that this may not happen".<br><br>"This is important if the resident has mental capacity if they have dementia it needs to be done with an advocate".
5.2 Were possible, with a resident's agreement, involve their family and friends as they can often provide valuable information	5.2 Where possible, with the consent of the person or their attorney, their family and friends should contribute to the care planning process as they can often provide valuable information.	"Consent should be gained to involve others or best interests decisions made if the person hasn't got mental capacity".<br><br>"Sometimes the family do not have the residents 'best interests' at heart".
5.3 Senior care or nursing staff are usually responsible for writing care plans; however, valuable information can also be provided by front line care workers and non-care staff - such as members of the housekeeping and catering teams.	5.3 Managers, senior care or nursing staff are usually responsible for writing care plans; as part of this process, valuable information can be collected from the wider care team, including front line care workers, and non-care staff, such as members of the housekeeping and catering teams.	"They are often missed out however have valuable insight from their own relationships built with the resident".<br><br>"It might be better to write that care plans are devised and written by those that are suitably trained with input from people across the team".
5.4 External health and care professionals, such as medical consultants, social workers, GPs, and occupational therapists, may contribute to specific parts of the care plan.	5.4 Information provided by external health and social care professionals - such as speech and language therapists, medical consultants, social workers, GPs, occupational therapists, physiotherapists, nurses and psychologists - may be added to specific parts of the care plan.	"Include physiotherapists, nurses, psychologists".<br><br>"It must be noted the time frame of professional's input to ensure it is relevant and up to date"
	**Section 6 - Who should have access to a care plan?**	
6.1 To be most useful, care plans will need to be accessible to:<br>• The residents themselves	6.1 If requested, a person should be provided with a copy of their care plan and provided with any additional information required to contextualise the plan's contents	"Must be guided or accompanied during their review of the care plan so the nurse can provide context to the contents of the document".<br><br>"Care plans are for the staff to use to look after the residents, residents can ask to look at them at anytime but would not normally have general access to them".
6.2 To be most useful, care plans will need to be accessible to:	6.2 If requested, a person's attorney should be provided with a copy of their care plan. When sharing details of a person's care	"You would not give an LPA for finances access to health information".

Original	Revised	Examples of feedback provided
• People who have legal power of attorney for the care home resident	plan, only information relevant to their inquiry should be disclosed.	"As and when required but this could be a third party solicitor and may not have any relevance to day to day care".
6.3 To be most useful, care plans will need to be accessible to:<br>• Members of a resident's "circle of care", such as named family and friends, that the resident or their Lasting Power of Attorney has consented to see their care plan/ certain sections of the care plan	6.3 If requested, and with the consent of the person or the appropriate attorney, members of a person's "circle of care", such as named family and friends, should be able to view their care plan. When sharing details of a person's care plan, only information relevant to a friend or family member's inquiry should be disclosed.	"Care Plans should not be open to multiple people to access unless the Resident themselves have consented to this".<br><br>"Not as needed as this would contain a lot of private information".<br><br>"Accessible but not constantly on offer".
6.4 To be most useful, care plans will need to be accessible to:<br>• Care home staff, including bank and agency staff	6.4 To be most useful, care plans will need to be accessible to:<br>• Care home staff, including bank and agency staff<br><br>Staff accessing a person's care plan must respect the principles of data protection.	"I assume there is already an assumption of confidentiality from all staff caring for residents. (…) A robust system of confidentiality is paramount".<br><br>"Bank staff must sign privacy and confidentiality agreement before access to these documents".
6.5 To be most useful, care plans will need to be accessible to:<br>• External health and care professionals, such as social workers, GPs, and pharmacists	6.5 When necessary to provide care, the relevant section of a person's care plan should be made accessible to external health and social care professionals – such as speech and language therapists, medical consultants, social workers, GPs, occupational therapists, physiotherapists, nurses and psychologists.<br><br>External professionals who view a person's care plan must respect the principles of data protection.	"Separate "GP Care Plans" are usually in place that are a short form, regularly updated and sent to the GP for notification of any changing needs etc".<br><br>"Depends on the person, their health and the issues. Also some parts of the care plan may not be relevant to some professionals". "Need to know basis under rules of confidentiality and data protection".
**Section 7 – Future Developments in care planning**		
7.1 Technology, such as digital care planning software, is playing an increasingly important role in supporting care planning. Digital care plans can:<br>• Help to reduce the amount of time to complete care plans	7.1 Technology, such as digital care planning software, is playing an increasingly important role in supporting care planning. Digital care plans can:<br>• Help to reduce the amount of time to complete and review care plans	"Having had digital software for over 8 years now, we have benefited greatly in the variety of care plans that we now complete and it reduces the length of time spent on these".<br><br>"I think they save time in reviewing care plan"
7.2 Technology, such as digital care planning software, is playing an increasingly important role in supporting care planning. Digital care plans can:<br>• Improve staff engagement in care planning	[Item removed]	*This statement has been removed because fewer than 75% of people who completed the survey rated this statement as "very important" or "extremely important"*<br><br>*People's comments indicate that no revision of this statement could make it acceptable to them*

Original	Revised	Examples of feedback provided
7.3 Technology, such as digital care planning software, is playing an increasingly important role in supporting care planning. Digital care plans can:<br>• Produce aggregate data which can help the home plan for the future	7.3 Technology, such as digital care planning software, is playing an increasingly important role in supporting care planning. Digital care plans can:<br>• Allow care homes to view information about all their residents, sometimes referred to as aggregate data, in order to help the home plan for the future.	"Data gathered can assist Care Homes with planning for staffing levels"<br><br>"This can help plan for staffing, training and audits"<br><br>"I'm not too sure what aggregate data is"
7.4 Technology, such as digital care planning software, is playing an increasingly important role in supporting care planning. Digital care plans can:<br>• Allow information to be securely and quickly shared with relevant stakeholders, such as health and social care professionals and a person's family and/or friends	7.4 Technology, such as digital care planning software, is playing an increasingly important role in supporting care planning. Digital care plans can:<br>• Where the appropriate consent has been given, allow information to be securely and quickly shared with relevant health and social care professionals and a person's family and/or friends	"Digital care plans are much easier to share with other professionals and family and friends. You can send via secure email or give families a code to access the care plans from home"<br><br>"Information can be quickly and securely emailed between agencies"<br><br>"With consent"
7.5 Care homes that are interested in adopting digital care plans may need to consider:<br>• Whether they have sufficient internet coverage across their site(s)	7.5 Care homes that are interested in adopting digital care plans may need to consider:<br>• Whether they have sufficient internet coverage across their site(s)	"A secure and safe internet connection is a must"<br><br>"It is essential that internet access is consistent and available to staff and families."
7.6 Care homes that are interested in adopting digital care plans may need to consider:<br>• The associated costs of software licences/updates, devices, network and data security, support and maintenance	7.6 Care homes that are interested in adopting digital care plans may need to consider:<br>• The associated costs of software licences/updates, devices, data security, backups, support and maintenance	"The cost of implementing digital care plans is more than just the actual software and hardware"<br>"Adopting digital care records is not a low-cost process. There are a number of additional costs over and above the cost of the DSCR software"
7.7 Care homes that are interested in adopting digital care plans may need to consider:<br>• If the software selected allows staff to develop person-centred care plans	7.7 Care homes that are interested in adopting digital care plans may need to consider:<br>• If the care planning software enables staff to develop person-centred care plans	"Correct. Software has to be chosen carefully."<br><br>"There could be a danger that drop-down boxes or shortcuts are built-in to speed up the process but are detrimental to the individualisation of the document"
7.8 Care homes that are interested in adopting digital care plans may need to consider:<br>• Whether the digital care plan can be made accessible to all the relevant people involved in supporting the resident, while ensuring that only appropriate people will be able to update the digital care plan	7.8 Care homes that are interested in adopting digital care plans may need to consider:<br>• Whether the digital care plan can be made accessible to all the relevant professionals involved in supporting the person, while ensuring that only appropriate people will be able to update the relevant section of the care plan	"Not all staff should be allowed to alter or update the care plans"<br><br>"Having different access levels is important to ensure that it's viewable by those required and being able to be editing by only those who should."
7.9 Care homes that are interested in adopting digital care plans may need to consider:<br>• The time commitment likely to be associated with transitioning from paper to digital care plans	7.9 Care homes that are interested in adopting digital care plans may need to consider:<br>• The time commitment associated with transitioning from paper to digital care plans	"Changing from paper to digital takes time to transfer this information"

Original	Revised	Examples of feedback provided
		“We have been surprised by the amount of work involved in the transfer to digital records and how long it takes”
7.10 Care homes that are interested in adopting digital care plans may need to consider:<br>• The time commitment likely to be associated with training and supporting staff to use digital care planning packages as well as meeting ongoing training needs	7.10 Care homes that are interested in adopting digital care plans may need to consider:<br>• The time commitment associated with training and supporting staff to use digital care planning packages as well as meeting ongoing training needs	“Selecting a system that is easy to onboard and for staff to use is very important”<br><br>“All staff need extensive training in the use of the systems. In my last 2 postings I have had no tech training and it has made my working life very difficult”.
