# Supplementary material for: Developing a Set of Key Principles for Care Planning Within Older Adult Care Homes: A Modified Delphi Survey
Source: Health Expect. 2025 Sep 29;28(5):e70433. doi: 10.1111/hex.70433 (PMC12477624; doi:10.1111/hex.70433)
Supplement: Supplementary file 5 — SI5‐Example‐of‐a‐panelists‐scores‐for‐each‐statement‐compared‐scores. [file HEX-28-e70433-s008.pdf]

Summary of section scores – Respondent ID: 1

Statement	Not at all important	Slightly important	Moderately important	Very important	Extremely important	I don't know	Your response was
1.1. What is the purpose of a care plan?<br><br>An effective care plan provides a snapshot of a resident's whole life, including their goals, skills, abilities and how they would like to manage their health and wellbeing.	0	1	4	19	78	0	Extremely important
1.2. What is the purpose of a care plan?<br><br>A strong care plan will acknowledge that a person's needs and interests can change over time, sometimes in response to changes to their health	0	0	2	21	79	0	Very important
1.3. What is the purpose of a care plan?<br><br>When done well, care plans will empower resident to have as much control and independence over their daily life as possible.	1	1	1	26	72	1	Very important
1.4. What is the purpose of a care plan?<br><br>The information contained within a strong care plan should help to:<br>• Identify residents' preferences and wishes each time staff provide care or support	0	0	3	16	83	0	Extremely important
1.5. What is the purpose of a care plan?<br><br>The information contained within a strong care plan should help to:<br>• Identify the views of residents or their family and friends, where possible, regarding the care and support they receive	0	1	7	30	64	0	Extremely important
1.6. What is the purpose of a care plan?<br><br>The information contained within a strong care plan should help to:<br>• Maintain continuity of care among external partners and collaborators	0	1	3	29	68	1	Extremely important
1.7. What is the purpose of a care plan?<br><br>The information contained within a strong care plan should help to:<br>• Assess resident's health and wellbeing over time	0	0	7	22	73	0	Extremely important
1.8. What is the purpose of a care plan?<br><br>The information contained within a strong care plan should help to:<br>• Assist in managing staffing levels and resources	2	5	21	38	34	2	Very important

Statement	Not at all important	Slightly important	Moderately important	Very important	Extremely important	I don't know	Your response was
1.9. What is the purpose of a care plan?<br><br>The information contained within a strong care plan should help to:<br>• Demonstrate that the identified care needs comply with quality-of-care standards	0	3	8	32	57	2	Extremely important
1.10. What is the purpose of a care plan?<br><br>The information contained within a strong care plan should help to:<br>• Set out what the resident's best life in the home would look like.	0	1	7	28	66	0	Very important
1.11. What is the purpose of an advanced care plan?<br><br>An effective advanced care plan will enable a care home resident to set out their preferences and priorities for future care.	0	0	0	22	80	0	Extremely important
1.12. What is the purpose of an advanced care plan?<br><br>Advanced care planning is designed to help ensure that the care that people receive in the future is consistent with their values, goals and preferences.	0	0	2	18	82	0	Extremely important
1.13. What is the purpose of an advanced care plan?<br><br>If not already in place, advanced care planning can lead to the appointment of a health and welfare Lasting Power of Attorney who is legally empowered to make decisions about the treatment a resident would receive if they no longer had the mental capacity to consent.	3	3	9	27	59	1	Moderately important
1.14. What is the purpose of an advanced care plan?<br><br>Advanced care plans often include information about a person's end of life care including where the person would like to die, if the person has completed a "do not attempt cardiopulmonary resuscitation" (DNACPR) form, and any, religious and/or spiritual requests.	0	0	1	17	84	0	Extremely important
1.15. What is the purpose of an advanced care plan?<br><br>Advanced care plans may also document a resident's future treatment preferences and where they would like to spend their last days.	0	0	1	22	78	1	Extremely important

Statement	Not at all important	Slightly important	Moderately important	Very important	Extremely important	I don’t know	Your response was
2.1. How can care planning be approached in a person centred way?<br><br>A person-centred care plan will help to meet a person's needs and preferences.	0	0	1	15	86	0	Very important
2.2. How can care planning be approached in a person centred way?<br><br>A person-centred care plan has the following qualities:<br><br>It provides a holistic understanding of a resident as an individual, including their history, current interests and future ambitions. It will detail:<br>• The social, emotional and health issues for which a resident requires support	0	1	0	23	77	1	Extremely important
2.3. How can care planning be approached in a person centred way?<br><br>A person-centred care plan has the following qualities:<br><br>It provides a holistic understanding of a resident as an individual, including their history, current interests and future ambitions. It will detail:<br>• The resident’s personal values and priorities for their care	0	0	1	25	75	1	Extremely important
2.4. How can care planning be approached in a person centred way?<br><br>A person-centred care plan has the following qualities:<br><br>It provides a holistic understanding of a resident as an individual, including their history, current interests and future ambitions. It will detail:<br>• The resident’s capabilities as well their needs	0	0	3	27	72	0	Very important
2.5. How can care planning be approached in a person centred way?<br><br>A person-centred care plan has the following qualities:<br><br>It engages the resident, and key stakeholders, in decision-making. This can be achieved by:<br>• Inviting residents to take the lead in discussing the care planâ€™s contents, wherever possible	0	0	8	23	70	1	Moderately important

2.6. How can care planning be approached in a person centred way?<br><br>A person-centred care plan has the following qualities:<br><br>It engages the resident, and key stakeholders, in decision-making. This can be achieved by:<br>• Taking reasonable steps to meet resident's communication (e.g., plain English, information available in Braille, translators) and sensory needs (e.g., hearing aids, glasses).	0	0	0	23	79	0	Extremely important
2.7. How can care planning be approached in a person centred way?<br><br>A person-centred care plan has the following qualities:<br><br>It engages the resident, and key stakeholders, in decision-making. This can be achieved by:<br>• Including input from important people in the resident's life	0	1	7	34	60	0	Very important
2.8. How can care planning be approached in a person centred way?<br><br>A person-centred care plan has the following qualities:<br><br>It engages the resident, and key stakeholders, in decision-making. This can be achieved by:<br>• Ensuring that residents are aware of all the available options and providing them with the information necessary to make informed decisions.	0	1	1	24	76	0	Very important
2.9. How can care planning be approached in a person centred way?<br><br>A person-centred care plan has the following qualities:<br><br>It engages the resident, and key stakeholders, in decision-making. This can be achieved by:<br>• Ensuring that with resident's consent*, family and friends can also be provided with the information necessary to make informed decisions to help them assist the resident in a making a decision.<br><br>* This consent may be delegated to a person granted Lasting Power of Attorney.	0	0	3	30	69	0	Very important

2.10. How can care planning be approached in a person centred way?<br><br>A person-centred care plan has the following qualities:<br><br>It engages the resident, and key stakeholders, in decision-making. This can be achieved by:<br>• Including input from external care providers, professionals and organisations involved in promoting the resident's health and wellbeing	0	1	10	29	62	0	Very important

Statement	Not at all important	Slightly important	Moderately important	Very important	Extremely important	I don't know	Your response was
3.1. What should be contained within a care plan?<br><br>Care plans will contain different sections. High quality care plans are likely to include:<br>• A recent photograph of the resident	0	1	9	17	75	0	Extremely important
3.2. What should be contained within a care plan?<br><br>Care plans will contain different sections. High quality care plans are likely to include:<br>• Details about the care plan itself:<br>○ A record of when the plan has been created, reviewed, updated and modified and when the care plan will next be reviewed.	0	1	3	24	74	0	Extremely important
3.3. What should be contained within a care plan?<br><br>Care plans will contain different sections. High quality care plans are likely to include:<br>• Background information about the resident's history, including details of:<br>○ The resident's life immediately prior to moving into the care home	0	1	7	34	60	0	Moderately important
3.4. What should be contained within a care plan?<br><br>Care plans will contain different sections. High quality care plans are likely to include:<br>• Background information about the resident's history, including details of:<br>○ The resident's family, culture and religion	0	0	4	23	75	0	Moderately important
3.5. What should be contained within a care plan?<br><br>Care plans will contain different sections. High quality care plans are likely to include:<br>• Background information about the resident's history, including details of:<br>○ Key dates and life events, such as significant holidays, anniversaries, and service honours	0	3	17	25	57	0	Moderately important

Statement	Not at all important	Slightly important	Moderately important	Very important	Extremely important	I don't know	Your response was
3.6. What should be contained within a care plan?<br><br>Care plans will contain different sections. High quality care plans are likely to include:<br>• Information about a resident's hobbies, interests and aspirations, past and present:<br>○ Information about how to support the resident's current goals	1	0	4	29	68	0	Moderately important
3.7. What should be contained within a care plan?<br><br>Care plans will contain different sections. High quality care plans are likely to include:<br>• Information about a resident's hobbies, interests and aspirations, past and present:<br>○ Information about activities the resident would/would not like to take part in and environments that they feel/do not feel comfortable in	1	1	9	22	69	0	Moderately important
3.8. What should be contained within a care plan?<br><br>Care plans will contain different sections. High quality care plans are likely to include:<br>• Information about the risks that the resident may face, and steps that can be taken to mitigate them in a person centred way	0	1	4	17	80	0	Extremely important
3.9. What should be contained within a care plan?<br><br>Care plans will contain different sections. High quality care plans are likely to include:<br>• Information about forthcoming appointments and details of who will be responsible for arranging transportation and accompanying the resident, these could be medical or social appointments	2	8	7	29	55	1	Very important
3.10. What should be contained within a care plan?<br><br>Care plans will contain different sections. High quality care plans are likely to include:<br>• Information about the resident's health, including, but not limited to:<br>○ Vital signs	0	0	8	23	70	1	Extremely important

Statement	Not at all important	Slightly important	Moderately important	Very important	Extremely important	I don't know	Your response was
3.11. What should be contained within a care plan?<br><br>Care plans will contain different sections. High quality care plans are likely to include:<br>• Information about the resident's health, including, but not limited to:<br>○ Medication	0	0	4	16	82	0	Very important
3.12. What should be contained within a care plan?<br><br>Care plans will contain different sections. High quality care plans are likely to include:<br>• Information about the resident's health, including, but not limited to:<br>○ Nutrition and hydration needs	0	0	3	15	84	0	Very important
3.13. What should be contained within a care plan?<br><br>Care plans will contain different sections. High quality care plans are likely to include:<br>• Information about the resident's health, including, but not limited to:<br>○ History of physical and mental health	0	1	1	18	82	0	Extremely important
3.14. What should be contained within a care plan?<br><br>Care plans will contain different sections. High quality care plans are likely to include:<br>• Information about the resident's day-to-day care needs and preferences, including:<br>○ The resident's capability to meet their own day-to-day needs and any preferences for receiving support	0	1	1	15	85	0	Extremely important
3.15. What should be contained within a care plan?<br><br>Care plans will contain different sections. High quality care plans are likely to include:<br>• Information about the resident's day-to-day care needs and preferences, including:<br>○ Details of any specialist equipment that the resident may need, such as adapted cutlery or hearing and mobility aids	0	0	0	14	88	0	Extremely important

Statement	Not at all important	Slightly important	Moderately important	Very important	Extremely important	I don't know	Your response was
3.16. What should be contained within a care plan?<br><br>Care plans will contain different sections. High quality care plans are likely to include:<br>• Information about a resident's end of life care, including:<br>○ Where the resident would like to be cared for	1	0	3	20	78	0	Extremely important
3.17. What should be contained within a care plan?<br><br>Care plans will contain different sections. High quality care plans are likely to include:<br>• Information about a resident's end of life care, including:<br>○ Details of religious, spiritual and/or cultural practices	1	0	2	20	79	0	Extremely important
3.18. What should be contained within a care plan?<br><br>Care plans will contain different sections. High quality care plans are likely to include:<br>• Information about a resident's end of life care, including:<br>○ Key people to involve	1	0	2	21	78	0	Extremely important
3.19. What should be contained within a care plan?<br><br>Care plans will contain different sections. High quality care plans are likely to include:<br>• Information about a resident's end of life care, including:<br>○ Who the resident would like to be with them in their final moments	1	1	3	21	75	1	Extremely important
3.20. What should be contained within a care plan?<br><br>Care plans will contain different sections. High quality care plans are likely to include:<br>• Information about a resident's end of life care, including:<br>○ Palliative medical care and resuscitation preferences	1	0	0	18	83	0	Extremely important
3.21. What should be contained within a care plan?<br><br>Care plans will contain different sections. High quality care plans are likely to include:<br>• Information about a resident's end of life care, including:<br>○ Funeral arrangements	3	1	10	25	62	1	Extremely important

3.22. What should be contained within a care plan?<br><br>Care plans will contain different sections. High quality care plans are likely to include:<br>• Information about a resident's end of life care, including:<br>○ Whether arrangements have been made for organ or body donations	1	3	11	22	62	3	Extremely important

Statement	Not at all important	Slightly important	Moderately important	Very important	Extremely important	I don't know	Your response was
4.1. When will a care plan be developed and updated?<br><br>A well-developed care plan will provide an accurate and up-to-date account of a resident's needs and interests.	1	0	2	13	86	0	Extremely important
4.2. When will a care plan be developed and updated?<br><br>Care plans should be thought of as a "live" document that will be continually updated.	0	1	1	12	88	0	Extremely important
4.3. When will a care plan be developed and updated?<br><br>Prior to, or shortly after, a person begins residence at a care home.	1	1	2	23	73	2	Extremely important
4.4. When will a care plan be developed and updated?<br><br>Where possible, key information about a resident (such as their health conditions and medical needs) should be included in a care plan prior to their admission to a care home.	1	0	3	21	77	0	Extremely important
4.5. When will a care plan be developed and updated?<br><br>This information - which could be gathered as part of a pre-admission assessment - may be obtained by talking to the resident, their General Practitioners (GP) or social worker and, with the resident's consent, their family and friends.	0	0	5	26	71	0	Very important
4.6. When will a care plan be developed and updated?<br><br>In the first 2-4 weeks following a person's arrival at a care home, as staff begin to get to know the resident better, it is often helpful to set aside time to develop a care plan.	1	2	3	30	66	0	Very important
4.7. When will a care plan be developed and updated?<br><br>A care plan should also be updated in response to significant changes or incidents in a resident's life, such as a fall, the development of new friendships, personal achievements, new hobbies and interests, a change in their health, or a hospital admission.	0	1	0	19	81	1	Very important
4.8. When will a care plan be developed and updated?<br><br>Thereafter, an effective care plan will be routinely updated, possibly in the form of regular and more meaningful and holistic reviews, to ensure the document reflects a resident's current needs and interests	0	1	3	28	69	1	Very important

Statement	Not at all important	Slightly important	Moderately important	Very important	Extremely important	I don’t know	Your response was
4.9. When will a care plan be developed and updated?<br><br>Where possible, and with a resident’s permission, family members may contribute to these reviews.	0	1	11	24	65	1	Very important
4.10. When will a care plan be developed and updated?<br><br>To ensure that care plans remain accurate and up-to-date, regular reviews are likely to take place at least every six weeks.	2	0	8	29	61	2	Extremely important
4.11. When will a care plan be developed and updated?<br><br>Regular reviews can provide an opportunity to assess the contents of a resident's care plan and discuss whether any changes need to be made.	0	1	5	25	70	1	Extremely important
4.12. When will a care plan be developed and updated?<br><br>More detailed care plan reviews may take place every six months	4	4	11	31	48	4	I don't know
Above, in statement 10, we have suggested that regular reviews of care plans are likely to take place at least every six weeks.<br><br>How often do you think these regular reviews should take place?	Once every four weeks - 64			Once every three weeks - 2			Once every four weeks
	Once every six weeks - 14			Once every two weeks - 2			
	Other - 8			Once a week - 1			
	Once every eight weeks - 6			Once every five weeks - 1			
	I don't know - 4						
Above, in statement 12, we have suggested that more detailed care plan reviews may take place every six months.<br><br>How often do you think these more detailed care plan reviews should take place?	Once every six months - 33			Once every two months - 5			Other - 12
	Once every three months - 24			I don't know - 4			
	Once a month - 17			Once every four months - 4			
	Other - 14			Once every eight months - 1			

Statement	Not at all important	Slightly important	Moderately important	Very important	Extremely important	I don’t know	Your response was
5.1. Who is likely to contribute to a care plan?<br><br>Where possible, residents should be involved in developing and reviewing their care plans.	0	0	3	12	87	0	Very important
5.2. Who is likely to contribute to a care plan?<br><br>Were possible, with a resident’s agreement, involve their family and friends as they can often provide valuable information.	0	1	8	23	70	0	Very important
5.3. Who is likely to contribute to a care plan?<br><br>Senior care or nursing staff are usually responsible for writing care plans; however, valuable information can also be provided by front line care workers and non-care staff - such as members of the housekeeping and catering teams.	0	0	16	17	69	0	Very important
5.4. Who is likely to contribute to a care plan?<br><br>External health and care professionals, such as medical consultants, social workers, GPs, and occupational therapists, may contribute to specific parts of the care plan.	0	1	10	24	66	1	Moderately important

Statement	Not at all important	Slightly important	Moderately important	Very important	Extremely important	I don't know	Your response was
6.1. Who should have access to a care plan?<br><br>To be most useful, care plans will need to be accessible to:<br>• The residents themselves	2	4	10	23	61	2	Very important
6.2. Who should have access to a care plan?<br><br>To be most useful, care plans will need to be accessible to:<br>• People who have legal power of attorney for the care home resident	0	1	7	37	55	2	Very important
6.3. Who should have access to a care plan?<br><br>To be most useful, care plans will need to be accessible to:<br>• Members of a resident's "circle of care", such as named family and friends, that the resident or their Lasting Power of Attorney has consented to see their care plan/ certain sections of the care plan	3	4	21	26	46	2	Moderately important
6.4. Who should have access to a care plan?<br><br>To be most useful, care plans will need to be accessible to:<br>• Care home staff, including bank and agency staff	0	1	0	15	86	0	Extremely important
6.5. Who should have access to a care plan?<br><br>To be most useful, care plans will need to be accessible to:<br>• External health and care professionals, such as social workers, GPs, and pharmacists	3	1	12	28	57	1	Extremely important

Statement	Not at all important	Slightly important	Moderately important	Very important	Extremely important	I don’t know	Your response was
7.1. Future developments in care planning<br><br>Technology, such as digital care planning software, is playing an increasingly important role in supporting care planning. Digital care plans can:<br>• Help to reduce the amount of time to complete care plans	3	3	12	37	44	3	Very important
7.2. Future developments in care planning<br><br>Technology, such as digital care planning software, is playing an increasingly important role in supporting care planning. Digital care plans can:<br>• Improve staff engagement in care planning	4	4	14	27	46	5	Very important
7.3. Future developments in care planning<br><br>Technology, such as digital care planning software, is playing an increasingly important role in supporting care planning. Digital care plans can:<br>• Produce aggregate data which can help the home plan for the future	3	3	9	35	44	7	Very important
7.4. Future developments in care planning<br><br>Technology, such as digital care planning software, is playing an increasingly important role in supporting care planning. Digital care plans can:<br>• Allow information to be securely and quickly shared with relevant stakeholders, such as health and social care professionals and a person’s family and/or friends	1	1	10	26	61	3	Extremely important
7.5. Future developments in care planning<br><br>Care homes that are interested in adopting digital care plans may need to consider:<br>• Whether they have sufficient internet coverage across their site(s)	2	2	1	23	71	2	Moderately important
7.6. Future developments in care planning<br><br>Care homes that are interested in adopting digital care plans may need to consider:<br>• The associated costs of software licences/updates, devices, network and data security, support and maintenance	2	4	9	33	51	2	Very important

7.7. Future developments in care planning<br><br>Care homes that are interested in adopting digital care plans may need to consider:<br>• If the software selected allows staff to develop person-centred care plans	2	1	4	23	67	4	Moderately important
7.8. Future developments in care planning<br><br>Care homes that are interested in adopting digital care plans may need to consider:<br>• Whether the digital care plan can be made accessible to all the relevant people involved in supporting the resident, while ensuring that only appropriate people will be able to update the digital care plan	0	1	6	23	69	2	Very important
7.9. Future developments in care planning<br><br>Care homes that are interested in adopting digital care plans may need to consider:<br>• The time commitment likely to be associated with transitioning from paper to digital care plans	1	5	12	25	57	2	Very important
7.10. Future developments in care planning<br><br>Care homes that are interested in adopting digital care plans may need to consider:<br>• The time commitment likely to be associated with training and supporting staff to use digital care planning packages as well as meeting ongoing training needs	1	7	7	25	61	1	Very important
