# Supplementary material for: Developing a Set of Key Principles for Care Planning Within Older Adult Care Homes: A Modified Delphi Survey
Source: Health Expect. 2025 Sep 29;28(5):e70433. doi: 10.1111/hex.70433 (PMC12477624; doi:10.1111/hex.70433)
Supplement: Supplementary file 7 — SI7‐List‐of‐round‐2‐statements‐and‐summary‐of‐respondents‐unipolar‐scores. [file HEX-28-e70433-s004.docx]

| **Statement** | **N** | **% "Very important" or "Extremely important"** | **Median** | **Mean** | **SD** |
| --- | --- | --- | --- | --- | --- |
| **1. What is the purpose of a(n advanced) care plan?** | | | | | |
| An effective care plan provides a summary of a person’s life, including their goals, skills, abilities and the support they need to manage their health and wellbeing. | 80 | 98.8% | 5 | 4.9 | 0.4 |
| A meaningful care plan will acknowledge that a person’s abilities, needs, interests and preferences, can change over time. | 80 | 97.5% | 5 | 4.9 | 0.4 |
| When done well, care plans will empower a person to have as much choice, control and independence over their daily life as possible. | 80 | 97.5% | 5 | 4.8 | 0.5 |
| The information contained within a meaningful care plan should help to:   - Identify a person's care needs, preferences and wishes each time staff provide support | 80 | 100.0% | 5 | 4.9 | 0.3 |
| The information contained within a meaningful care plan should help to:   - Identify the views of the person regarding the care and support they receive. It can be helpful to supplement these with the views of family and friends. Wherever possible, the views of the person receiving care should be prioritised | 80 | 97.5% | 5 | 4.7 | 0.5 |
| The information contained within a meaningful care plan should help to:   - Maintain continuity of care among other health and social care professionals – such as GPs, district nurses and physiotherapists - involved in an individual’s care | 80 | 97.5% | 5 | 4.7 | 0.5 |
| The information contained within a meaningful care plan should help:   - Staff to assess and monitor a person’s health and wellbeing over time | 80 | 92.5% | 5 | 4.6 | 0.7 |
| The information contained within a meaningful care plan should help to:   - Give a clearer indication of a person’s needs. This information may help to inform the resources and staffing levels required in the care home | 80 | 91.3% | 5 | 4.5 | 0.7 |
| The information contained within a meaningful care plan should help to:   - Demonstrate that the care that a person receives complies with the relevant CQC and NICE guidelines | 80 | 88.8% | 5 | 4.5 | 0.8 |
| The information contained within a meaningful care plan should help:   - Staff to support a person to live a fulfilling life | 80 | 97.5% | 5 | 4.8 | 0.4 |
| An effective advance care plan will enable a person to set out their preferences and priorities for future care, including end of life care. | 80 | 100.0% | 5 | 4.9 | 0.4 |
| An advance care plan is designed to help ensure that the care that people receive in the future is consistent with their values, goals and preferences. | 80 | 97.5% | 5 | 4.8 | 0.5 |
| If not already in place, advance care planning can lead to the appointment of an Attorney under the terms of a Lasting Power of Attorney or a Court of Protection Deputy who is empowered to make decisions on behalf of the person. | 80 | 88.8% | 5 | 4.4 | 1.0 |
| Advance care plans often include information about a person’s end of life care including where the person would like to die, if the person has completed a “do not attempt cardiopulmonary resuscitation” (DNACPR) or ReSPECT form, and any religious and/or spiritual requests. | 80 | 93.8% | 5 | 4.8 | 0.6 |
| Advance care plans may also document a person’s future treatment preferences and where and how they would like to spend their last days. | 80 | 100.0% | 5 | 4.8 | 0.4 |
| **2. How can care planning be approached in a person-centred way?** | | | | | |
| Person centred care planning affirms who a person is, their dignity, and prioritises their individual needs and wishes over and above generic routines and institutional practices. | 80 | 98.8% | 5 | 4.7 | 0.7 |
| Where appropriate, person-centred care planning may involve consulting with a person’s attorney and other important people in their lives. | 80 | 97.5% | 5 | 4.5 | 0.5 |
| A person-centred care plan can: ensure that a person's voice is heard, and their wishes inform the care they receive | 80 | 98.8% | 5 | 4.8 | 0.4 |
| A person-centred care plan can:   - Empower a person | 80 | 95.0% | 5 | 4.6 | 0.6 |
| A person-centred care plan can:   - Build trusting relationships between the person and their care team | 80 | 97.5% | 5 | 4.7 | 0.5 |
| A person-centred care plan will help to ensure that a person's abilities are supported, and their wishes, preferences and needs are met. | 80 | 98.8% | 5 | 4.8 | 0.4 |
| It provides a complete picture of who the person is, including their history, current interests and future ambitions. It will detail:   - The social, cultural, behavioural, environmental, emotional and health needs for which a person requires support | 80 | 100.0% | 5 | 4.8 | 0.4 |
| It provides a complete picture of who the person is, including their history, current interests and future ambitions. It will detail:   - The person’s beliefs, values and what is important to them | 80 | 98.8% | 5 | 4.8 | 0.5 |
| A person-centred care plan has the following qualities:   - It provides a complete picture of who the person is, including their history, current interests and future ambitions. It will detail:   - The person’s abilities, to empower their independence and foster a sense of purpose, belonging and self-esteem | 80 | 98.8% | 5 | 4.7 | 0.5 |
| A person-centred care plan has the following qualities:   - It engages the person in decision-making. This can be achieved by:   - Inviting the person to describe the support that they would like to receive so they are central to their care choices | 80 | 98.8% | 5 | 4.8 | 0.4 |
| A person-centred care plan has the following qualities:   - It engages the person in decision-making. This can be achieved by:   - Taking reasonable steps to communicate in a way that the person can understand. This may involve using specialised visual, language, auditory, sensory tools - such as Braille and translators - as well as staff insights into a person’s specific communication methods | 80 | 98.8% | 5 | 4.8 | 0.4 |
| A person-centred care plan has the following qualities:   - It engages the person in decision-making. This can be achieved by:   - Inviting, where appropriate, the person, or their attorney, to consent to important people in their life - who know them well and understand their individual needs and wishes and will act in their best interests - providing additional information | 80 | 95.0% | 5 | 4.6 | 0.6 |
| A person-centred care plan has the following qualities:   - It engages the person in decision-making. This can be achieved by:   - Ensuring that, with the consent of the person or their attorney, important people in their life can be provided with the information necessary to help them make decisions | 80 | 96.3% | 5 | 4.6 | 0.6 |
| A person-centred care plan has the following qualities:   - It engages the person in decision-making. This can be achieved by:   - Ensuring that the person is aware of all the available options and providing them with the information necessary to make informed decisions | 80 | 97.5% | 5 | 4.8 | 0.5 |
| A person-centred care plan has the following qualities:   - It engages the person in decision-making. This can be achieved by:   - Including input from external health and social care professionals that will support the person's wellbeing, without disregarding the person’s wishes and preferences | 80 | 97.5% | 5 | 4.6 | 0.5 |
| **3. What should be contained within a care plan?** | | | | | |
| Care plans will contain different sections. High quality care plans are likely to include:   - A recent dignified photograph of the person, which will be updated regularly | 80 | 90.0% | 5 | 4.5 | 0.7 |
| Care plans will contain different sections. High quality care plans are likely to include:   - Details about the care plan itself:   - A record of when the plan was created, revised and will next be reviewed and who is responsible for providing particular care | 80 | 91.3% | 5 | 4.5 | 0.7 |
| Care plans will contain different sections. High quality care plans are likely to include:   - Background information about the person's history, including details of:   - The person’s life immediately prior to moving into the care home and routines that were important to them | 80 | 96.3% | 5 | 4.6 | 0.6 |
| Care plans will contain different sections. High quality care plans are likely to include:   - Background information about the person's history, including details of:   - The person's gender identity, sexuality, family, culture and religion | 80 | 93.8% | 5 | 4.5 | 0.7 |
| Care plans will contain different sections. High quality care plans are likely to include:   - Background information about the person's history, including details of:   - Key dates and life events, such as significant holidays, anniversaries, volunteering and service honours and the support required from the home to enable the person to celebrate these milestones | 80 | 91.3% | 5 | 4.5 | 0.7 |
| Care plans will contain different sections. High quality care plans are likely to include:   - Information about a person's hobbies, interests, achievements and aspirations, past and present:   - Information about how to support the person to pursue these interests | 80 | 95.0% | 5 | 4.6 | 0.6 |
| Care plans will contain different sections. High quality care plans are likely to include:   - Information about a person's hobbies, interests, achievements and aspirations, past and present:   - Information about activities the person would/would not like to take part in and environments that they feel/do not feel comfortable in | 80 | 91.3% | 5 | 4.5 | 0.7 |
| Care plans will contain different sections. High quality care plans are likely to include:   - Information about the key risks that the person may face, and steps that can be taken to keep them safe in the least restrictive way possible | 80 | 97.5% | 5 | 4.7 | 0.5 |
| Care plans will contain different sections. High quality care plans are likely to include:   - Information about forthcoming appointments – such as medical or social appointments – and the support that the care home should provide to enable these to take place | 80 | 92.5% | 5 | 4.5 | 0.8 |
| Care plans will contain different sections. High quality care plans are likely to include:   - Information about the person's health, including, but not limited to:   - Vital signs, which can provide an important baseline to compare against in the future | 80 | 97.5% | 5 | 4.7 | 0.5 |
| Care plans will contain different sections. High quality care plans are likely to include:   - Information about the person's health, including, but not limited to:   - Medication | 80 | 100.0% | 5 | 4.8 | 0.4 |
| Care plans will contain different sections. High quality care plans are likely to include:   - Information about the person's health, including, but not limited to:   - The person’s nutrition and hydration needs and preferences | 80 | 98.8% | 5 | 4.8 | 0.4 |
| Care plans will contain different sections. High quality care plans are likely to include:   - Information about the person's health, including, but not limited to:   - Their physical, emotional and social wellbeing | 80 | 98.8% | 5 | 4.8 | 0.5 |
| Care plans will contain different sections. High quality care plans are likely to include:   - Information about the person's day-to-day care needs and preferences, including:   - The person’s capability to meet their own day-to-day needs and their preferences for how support should be delivered | 80 | 98.8% | 5 | 4.8 | 0.4 |
| Care plans will contain different sections. High quality care plans are likely to include:   - Information about the person's day-to-day care needs and preferences, including:   - Details of any equipment that the person uses, associated staff training needs, and how the equipment should be maintained | 80 | 96.3% | 5 | 4.8 | 0.5 |
| Care plans will contain different sections. Advanced care plans, which will form part of the wider care plan, should include:   - Information about a person's end of life care, including:   - Where the person would like to be cared for | 80 | 97.5% | 5 | 4.7 | 0.5 |
| Care plans will contain different sections. Advanced care plans, which will form part of the wider care plan, should include: Information about a person's end of life care, including: Details of religious, spiritual and/or cultural practices | 80 | 96.3% | 5 | 4.7 | 0.5 |
| Care plans will contain different sections. Advanced care plans, which will form part of the wider care plan, should include:   - Information about a person's end of life care, including:   - Key people to involve | 80 | 98.8% | 5 | 4.7 | 0.7 |
| Care plans will contain different sections. Advanced care plans, which will form part of the wider care plan, should include:   - Information about a resident’s end of life care, including:   - Who the person would like to be with them in their final moments, either in-person or virtually using software such as MS Teams, WhatsApp or FaceTime | 80 | 95.0% | 5 | 4.6 | 0.9 |
| Care plans will contain different sections. Advanced care plans, which will form part of the wider care plan, should include:   - Information about a resident’s end of life care, including:   - Palliative medical care and resuscitation preferences which may also be documented using a ReSPECT form | 80 | 98.8% | 5 | 4.8 | 0.4 |
| Care plans will contain different sections. Advanced care plans, which will form part of the wider care plan, should include:   - Information about a person's end of life care, including:   - Funeral arrangements | 80 | 91.3% | 5 | 4.5 | 0.7 |
| Care plans will contain different sections. Advanced care plans, which will form part of the wider care plan, should include:   - Information about a person's end of life care, including:   - Whether arrangements have been made for organ or body donations | 80 | 92.5% | 5 | 4.6 | 0.7 |
| **4. When will a care plan be developed and updated?** | | | | | |
| A care plan should be regularly reviewed, and updated, if necessary, to provide an accurate account of how to care for a person safely while meeting their needs and supporting their interests and preferences. | 80 | 100.0% | 5 | 4.9 | 0.3 |
| To provide safe and person-centred care from the outset, it is very important to gather as much information as possible prior to person’s arrival in a care home. This information should include a person’s needs, interests and preferences and can be used as the foundation for their care plan. | 80 | 96.3% | 5 | 4.8 | 0.6 |
| The information collected prior to a person’s arrival may be obtained by talking to them and, with their consent or that of their attorney, contacting previous care settings, health and social care professionals, and their family and friends. | 80 | 93.8% | 5 | 4.6 | 0.6 |
| Shortly after a person’s arrival, as staff begin to get to know them better, it is important to set aside time to update their initial care plan. | 80 | 97.5% | 5 | 4.8 | 0.5 |
| An effective care plan will be routinely reviewed to ensure the document reflects a person’s current needs, interests, and preferences. Reviews can take two forms: (1) in response to changes and/or (2) in accordance with a prearranged timeframe. | 80 | 100.0% | 5 | 4.7 | 0.5 |
| A care plan should be updated in response to changes in a person’s life. These changes could relate to their health, social or emotional wellbeing, interests and preferences, a hospital admission, the death of an important person in their life, their goals and ambitions, abilities and newly identified safety risks. | 80 | 100.0% | 5 | 4.8 | 0.4 |
| If no changes have been observed, prearranged reviews should take place once every four to six weeks to ensure that care plans remain accurate and up-to-date. | 80 | 90.0% | 5 | 4.5 | 0.9 |
| Where possible, and with a person’s consent or that of their attorney, significant people in their lives, such as family or friends, may contribute to prearranged review. | 80 | 91.3% | 4.5 | 4.4 | 0.6 |
| Prearranged reviews, which should involve the person and, if applicable, their attorney, will provide an opportunity to assess the contents of their care plan, identify any changes in their care needs and document how these will be met. | 80 | 90.0% | 5 | 4.5 | 0.7 |
| **5. Who is likely to contribute to a care plan?** | | | | | |
| A person must be involved in developing and reviewing their care plans. The manner of a person’s involvement will be informed by their capacity – for example, people with limited capacity may require a proxy to assist them. | 80 | 93.8% | 5 | 4.6 | 0.8 |
| Where possible, with the consent of the person or their attorney, their family and friends should contribute to the care planning process as they can often provide valuable information. | 80 | 93.8% | 5 | 4.5 | 0.6 |
| Managers, senior care or nursing staff are usually responsible for writing care plans; as part of this process, valuable information can be collected from the wider care team, including front line care workers, and non-care staff, such as members of the housekeeping and catering teams. | 80 | 93.8% | 5 | 4.5 | 0.7 |
| Information provided by external health and social care professionals - such as speech and language therapists, medical consultants, social workers, GPs, occupational therapists, physiotherapists, nurses and psychologists - may be added to specific parts of the care plan. | 80 | 93.8% | 5 | 4.5 | 0.6 |
| **6. Who should have access to a care plan?** | | | | | |
| If requested, a person should be provided with a copy of their care plan and provided with any additional information required to contextualise the plan’s contents. | 80 | 88.8% | 5 | 4.5 | 0.8 |
| If requested, a person’s attorney should be provided with a copy of their care plan. When sharing details of a person’s care plan, only information relevant to their inquiry should be disclosed. | 80 | 88.8% | 4 | 4.3 | 0.8 |
| To be most useful, care plans will need to be accessible to care home staff, including bank and agency staff.  Staff accessing a person’s care plan must respect the principles of data protection | 80 | 97.5% | 5 | 4.8 | 0.4 |
| When necessary to provide care, the relevant section of a person’s care plan should be made accessible to external health and social care professionals – such as speech and language therapists, medical consultants, social workers, GPs, occupational therapists, physiotherapists, nurses and psychologist.  External professionals who view a person’s care plan must respect the principles of data protection. | 80 | 93.8% | 5 | 4.5 | 0.6 |
| **7. Future developments in care planning** | | | | | |
| Technology, such as digital care planning software, is playing an increasingly important role in supporting care planning. Digital care plans can:   - Help to reduce the amount of time to complete and review care plans | 80 | 78.8% | 4 | 4.1 | 0.9 |
| Technology, such as digital care planning software, is playing an increasingly important role in supporting care planning. Digital care plans can:   - Allow care homes to view information about all their residents, sometimes referred to as aggregate data, in order to help the home plan for the future | 80 | 86.3% | 4 | 4.3 | 0.7 |
| Technology, such as digital care planning software, is playing an increasingly important role in supporting care planning. Digital care plans can:   - Where the appropriate consent has been given, allow information to be securely and quickly shared with relevant health and social care professionals and a person’s family and/or friends | 80 | 86.3% | 5 | 4.5 | 0.7 |
| Care homes that are interested in adopting digital care plans may need to consider:   - Whether they have sufficient internet coverage across their site(s) | 80 | 91.3% | 5 | 4.6 | 0.6 |
| Care homes that are interested in adopting digital care plans may need to consider:   - The associated costs of software licences/updates, devices, data security, backups, support and maintenance | 80 | 86.3% | 5 | 4.4 | 0.9 |
| Care homes that are interested in adopting digital care plans may need to consider:   - If the care planning software enables staff to develop person-centred care plans | 80 | 92.5% | 5 | 4.6 | 0.6 |
| Care homes that are interested in adopting digital care plans may need to consider:   - Whether the digital care plan can be made accessible to all the relevant professionals involved in supporting the person, while ensuring that only appropriate people will be able to update the relevant section of the care plan | 80 | 87.5% | 5 | 4.5 | 0.8 |
| Care homes that are interested in adopting digital care plans may need to consider:   - The time commitment associated with transitioning from paper to digital care plans | 80 | 83.8% | 5 | 4.3 | 0.8 |
| Care homes that are interested in adopting digital care plans may need to consider:   - The time commitment associated with training and supporting staff to use digital care planning packages as well as meeting ongoing training needs | 80 | 85.0% | 5 | 4.5 | 0.8 |
